# A computational model for the cancer field effect

**DOI:** 10.3389/frai.2023.1060879

**Published:** 2023-07-04

**Authors:** Karl Deutscher, Thomas Hillen, Jay Newby

**Affiliations:** Department of Mathematical and Statistical Sciences, University of Alberta, Edmonton, AB, Canada

**Keywords:** cancer field effect, field cancerization, carcinogenesis, head and neck squamous cell carcinoma, computational modeling, hybrid cellular automaton, smoking

## Abstract

**Introduction:**

The Cancer Field Effect describes an area of pre-cancerous cells that results from continued exposure to carcinogens. Cells in the cancer field can easily develop into cancer. Removal of the main tumor mass might leave the cancer field behind, increasing risk of recurrence.

**Methods:**

The model we propose for the cancer field effect is a hybrid cellular automaton (CA), which includes a multi-layer perceptron (MLP) to compute the effects of the carcinogens on the gene expression of the genes related to cancer development. We use carcinogen interactions that are typically associated with smoking and alcohol consumption and their effect on cancer fields of the tongue.

**Results:**

Using simulations we support the understanding that tobacco smoking is a potent carcinogen, which can be reinforced by alcohol consumption. The effect of alcohol alone is significantly less than the effect of tobacco. We further observe that pairing tumor excision with field removal delays recurrence compared to tumor excision alone. We track cell lineages and find that, in most cases, a polyclonal field develops, where the number of distinct cell lineages decreases over time as some lineages become dominant over others. Finally, we find tumor masses rarely form via monoclonal origin.

## 1. Introduction

The idea of field cancerization was first mentioned by Slaughter et al. (1953) in 1953 when histologically observing 783 squamous-cell tumors in oral cancers. Within the entire patient population it was found that benign epithelium surrounding the malignant tumor was abnormal. As well, some of the patients had multiple separate tumors occur in the same area of the oral cavity. From these observations, Slaughter et al. proposed a process termed *field cancerization*, in which a carcinogenic agent preconditions an area of epithelium toward cancer. If an area of epithelium is exposed to a carcinogenic agent for a sufficient amount of time and with enough intensity then it produces irreversible changes in cells and cell groups, such that the process toward cancer becomes inevitable (Slaughter et al., 1953). In Slaughter et al. (1953), it is also noted that a field of preconditioned epithelium may develop cancer at multiple points and possibly lead to multiple tumors. As a result, cancer does not arise from one cell that suddenly becomes malignant but instead from areas of precancerous change. Local recurrence after surgery or radiation occurs due to left-over benign epithelium that is preconditioned toward cancer, i.e., from the remaining pre-cancer field. Many papers were written following (Slaughter et al., 1953) that showed field cancerization can be found in colon carcinoma (Galandiuk et al., 2012; Alonso et al., 2015), gastric carcinoma (Kang et al., 1997; Zaky et al., 2008; Takeshima et al., 2015), esophageal carcinoma (Cense et al., 1997; Oka et al., 2009; Lee et al., 2011), non-small-cell lung squamous carcinoma (Franklin et al., 1997; Steiling et al., 2008; Kadara and Wistuba, 2012), non-small-cell lung adenocarcinoma (Gomperts et al., 2013; Kadara et al., 2014), head and neck squamous cell carcinoma (HNSCC) (oral, oropharynx, hypopharynx, larynx) (Slaughter et al., 1953; Califano et al., 1996; Braakhuis et al., 2003; Angadi et al., 2012), breast carcinoma (Trujillo et al., 2011; Rivenbark and Coleman, 2012; Foschini et al., 2013), cervix (Chu et al., 1999), prostate carcinoma (Nonn et al., 2009; Trujillo et al., 2012), bladder carcinoma (Hafner et al., 2002), and skin carcinoma (Kanjilal et al., 1995; Hu et al., 2012; Szeimies et al., 2012).

Biomarkers that were discovered to correlate with the presence of a cancer field are loss of heterozygosity (LOH) (Tabor et al., 2001), micro-satellite alterations (Tabor et al., 2001), chromosomal instability (Hittelman, 2001), and mutations in the TP53 gene (Brennan et al., 1995; van Houten et al., 2002). Braakhuis et al. (2003) related field cancerization to genetics and identified: “*growth of one or more genetically altered cell(s) that produces a field of cells predisposed to subsequent tumor growth*.” Based on genetic evidence, there currently exists two main hypotheses that explain the underlying cellular basis of field cancerization: polyclonal origin and monoclonal origin. Polyclonal origin proposes that mutations occur in multiple sites of the epithelium due to continuous carcinogen exposure, which leads to multi-focal carcinomas or lesions of independent origin (van Oijen and Slootweg, 2000). Monoclonal origin proposes that the mutant cells from the initial lesion migrate and develop multiple lesions that share a common clonal origin (Braakhuis et al., 2003). Here, we show that while all cases are possible in our model, a polyclonal field is by far the most common outcome of our simulations.

Another breakthrough in biology since (Slaughter et al., 1953) was the discovery of cancer stem cells CSCs and their importance in cancer initiation, progression, and treatment. Simple et al. (2015) explain field cancerization using Braakhuis model of genetic alterations (Braakhuis et al., 2003) plus the addition of CSCs. They consider both monoclonal and polyclonal origin within their model. In Simple's model (Simple et al., 2015) for oral cancer, a continuous exposure of the oral mucosa to carcinogens results in molecular alterations that lead to the induction of CSC-like behavior in a step-wise manner. CSCs originate either by transformation of normal stem cells (NSCs), or by dedifferentiation of the tumor cells (TC) and migration through normal mucosa to develop the field. Repeated mutations at 17p (the location of the TP53 gene) and 3p/9p (p16/FHIT gene) lead to transformation of the NSCs into transit amplifying cells (TACs). These transformed cells divide and expand to create a field of neoplastic cells. Finally, a genetic hit in the cells within the field at 13q (the location of the Rb gene) allows a carcinoma to develop. Note that alteration to the Rb gene is known to release CSCs from their quiescent stage such that proliferation, self-renewal and formation of tumors can occur. The work of Simple motivated us to include gene expression levels explicitly in our model, and to use a neural network to describe those changes.

Recently Curtius et al. (2017) studied field cancerization from an evolutionary perspective. They define a cancerized field to be a single cell or group of cells that are further along the evolutionary path toward cancer. Driver mutations have been found in both the carcinoma and the cancerized field thus indicating that a driver mutation may also be a field cancerization characteristic. As a result, field cancerization can occur because of multiple independent clonal expansions, i.e., polyclonal origin. Thus, both Simple et al. (2015) and Curtius et al. (2017) consider that a cancerized field can be formed via monoclonal or polyclonal origin.

### 1.1. Our model

In our model, a cancer field will be considered as a region of tissue that has genetic and phenotypic change that preconditions it toward the possible formation of one or more tumors within it. The genetic and phenotypic change can be caused by carcinogenic onslaught, genetic defects at birth, mutations later in life, or a combination thereof. We focus on the effect of a certain body region or organ—such as the mouth or the tongue—and we do not consider a system-wide pre-mutation as a cancer field.

The steps of the process of field cancerization that will be considered here are as follows:

- A region of tissue is repeatedly affected by one or more carcinogens over time, for example through smoking tobacco.
- The carcinogen(s) cause genetic mutations in the cells of the tissue which in turn influence the phenotype of the cell;
- As the cells start to proliferate and differentiate, the field expands;
- Eventually a CSC will be created, which will finally create the first TC and consequently a tumor.

We develop the model in the framework of a hybrid cellular automaton as introduced by Gerlee and Anderson (2007). The details will be explained in Section 2. With this model we try to answer:

1. What degree of carcinogenic onslaught is necessary for field cancerization to occur? Which carcinogens are the most aggressive, smoking related carcinogens or alcohol?
2. How long before a cancer field is formed? How long before the first tumor cell within the field is formed?
3. Is the field formed via monoclonal origin, polyclonal origin, or a mixture of both? Which type of origin is the most common?
4. How long does it take for a tumor to be large enough such that it is detectable by physicians? Once a tumor is detected, what size is the surrounding field?
5. How long does it take a recurrence to occur after removal of the tumor vs. the removal of tumor and field?
6. What are the dynamics of different cell lineages in an established cancer field?

Though field cancerization is found in many types of tissue throughout the body, the most commonly studied case is head and neck squamous cell carcinoma (HNSCC), which we consider here. Alcohol and smoking are the most commonly associated carcinogens to HNSCC. These two carcinogens typically enter the body through smoking and/or chewing tobacco and drinking alcohol, respectively. We use parameter values that are typically associated to smoking and alcohol drinking.

We find in our model, that a continued, long-time onslaught of carcinogen on tissue of the mouth inevitably leads to a formation of a cancer field. Our model confirms the general understanding (Hashibe et al., 2009; LoConte et al., 2018) that smoking induced carcinogens are much more potent than alcohol. The timing of the first tumor cell is usually quite long, in the order of 10s of years, hence for many people, a cancer will not arise. In almost all situations the field is polyclonal. Monoclonal fields are only seen in very small domains. A removal of the tumor, in our model, leads to a quick recurrence if the field is left behind. If the field is removed as well, then recurrence takes very long time. When we follow the cell lineages, we see that new lineages form and several of them will die out over time. However, some lineages establish themselves and become cancerous. In that case we observe a polyclonal cancer field, and also polyclonal cancers.

### 1.2. Carcinogenesis

Most carcinogenesis models consider that cancer is initialized from the result of a multi-step process (Frank, 2007). A normal cell does not become a cancer cell until multiple genetic alterations accumulate within it. The number of genetic alterations in a cancer cell is an indicator of the level of malignancy of the cell.

Gatenby and Gillies (2008) found six micro-environmental barriers for a malignant phenotype: apoptosis with loss of basement membrane contact, inadequate growth promotion, senescence (deterioration of a cells' power of division and growth with age), hypoxia (deficiency in the amount of oxygen reaching the tissues), acidosis (excessively acidic condition of the body fluids or tissues), and ischaemia (restriction of blood supply to tissues, causing hypoxia). The development of cancer occurs when a normal cell overcomes at least one of these barriers. Thus, the micro-environment is an important factor to consider in cancer initialization.

A normal cell lineage can acquire mutations (Curtius et al., 2017), that are positively selected in the micro-environment of a healthy organ. A driver mutation is one that confers growth or survival advantages for tumor cells within the appropriate micro-environment (Greaves et al., 2003; Calabrese et al., 2004; Stratton et al., 2009). A passenger (neutral) mutation is one that passively accumulates in cell lineages (Greaves et al., 2003; Calabrese et al., 2004; Stratton et al., 2009). It may be that some driver mutations are not currently affecting cancer growth but instead had previously driven the growth of an lineage (Curtius et al., 2017). Progression to cancer usually requires the accumulation of multiple driver mutations (Weaver et al., 2014). A mutant lineage/clone, can grow to produce large patches, or fields, of cells that are predisposed to eventually progress to neoplasm.

It has been reported by Knopf et al. (2015) that at least 232 genes are directly involved in HNSCC of young patients. Here, we focus on 10 genes of importance: TP53, TP73, RB, TP21, T16, EGFR, CCDN1, MYC, PIK3CA, and RAS (Knopf et al., 2015). Some of these genes are oncogenes (EGFR, CCDN1, MYC, PIK3CA, RAS), i.e., supporting cancer development if over expressed, and some are tumor suppressor genes (TP53, TP73, RB, TP21, TP16), i.e., support tumor growth if expression is inhibited.

### 1.3. Cancer stem cells

Before discussing cancer stem cells (CSCs) it should be noted that there is no single standardized definition of CSCs. Instead, many slightly different and sometimes contradictory definitions have emerged, each suited to a particular study. In general, CSCs are not normal stem cells (NSC), they are cells that have some of the characteristics of NSC. We consider CSCs as multipotent cells in a tumor that like NSCs have self-renewal ability, but in addition, have the abilities of tumor initiation, migration and metastasis (Biddle et al., 2011; Bu and Cao, 2012).

The origin of CSCs is explained by three possible processes. The first process states that a NSC undergoes several genetic as well as epigenetic alterations to give rise to a CSC (Feller et al., 2013). The second process states that CSCs originate from NSCs that acquire a precancerous phenotype during their development stage (Bjerkvig et al., 2005; Feller et al., 2013; González-Moles et al., 2013). The third process states that the CSC originate from mature tumor cells (Moon et al., 2011; Kumar et al., 2012; Herreros-Villanueva et al., 2013; Di Fiore et al., 2014) or epithelial cells (Bjerkvig et al., 2005; Feller et al., 2013; González-Moles et al., 2013) that undergo dedifferentiation into a CSC through modifications in signaling pathways and regulatory mechanisms. Note that the first and second processes only differ in whether an NSC acquires a genetic alteration when it is fully developed or still in development.

### 1.4. Previous mathematical models

There exists an extensive amount of literature that studies cancer initiation (Gentry and Jackson, 2013; Durrett et al., 2016; Paterson et al., 2020), progression (Beerenwinkel et al., 2014; Enderling and Chaplain, 2014), metastasis (Franssen et al., 2019), treatment (chemotherapy, immunotherapy, radiation) (de Pillis et al., 2009; Enderling and Chaplain, 2014; Radunskaya et al., 2018), and effects of various micro-environmental and external factors on cancer development (Gerlee and Anderson, 2007). However, the only mathematical model for field cancerization, that could be found at the time of writing, is the model by Foo et al. and its follow-up studies (Foo et al., 2014, 2020, 2022; Ryser et al., 2016). Here, we will further extend Foo's model using the hybrid cellular automaton approach of Gerlee and Anderson (2007).

Foo et al. (2014) describe field cancerization as a spatial Moran process on a square lattice. The cells are classified into three phenotypes, *k* = 0 healthy, *k* = 1 pre-cancerous, *k* = 2 cancer cells. These phenotypes have different fitness, with healthy having the lowest fitness, pre-cancer cells an intermediate fitness, and cancer cells the highest fitness. Random mutations allow cells to transit from *k* = 0 to *k* = 1 and *k* = 2. Foo et al. (2014) consider the dependence of their model on the parameters, and they identify three regimes. A first regime where pre-cancer cells quickly become cancerous. In that case no field is generated, as the progression to cancer is fast. A second regime where progress from pre-cancerous to cancerous is slow. This leads to a significant sized cancer field and multiple lesions in the tissue. And a third regime, where Field development and cancer development are on the same time scale. More recently, Foo et al. (2020, 2022) extended the model to include a three dimensional tissue structure consisting of epithelial layers, and more than *k* = 2 phenotypes.

We use these models by Foo et al.'s (2014, 2020, 2022) as a starting point for our model and we extend it by (i) allowing multiple mutations at different genes, which can or cannot lead to cancer development, (ii) allow for non-constant micro-environments, (iii) consider carcinogens as mutational driver, (iv) follow cell lineages, and consider additional phenotypic action such as apoptosis, quiescence, transit amplifying cells, and de-differentiation.

Ryser et al. (2016) applied the model described in Foo et al. (2014) to head and neck squamous cell carcinoma (HNSCC). The three histopathological stages of epithelial dysplasia (precancerous stages) are mild, moderate, and severe (carcinoma *in situ* [CIS]). Ryser et al. (2016) consider the following four type of cells: normal cells (type 0), mildly dysplastic cells (type 0*), moderately dysplastic cells (type 1), and severely dysplastic cells (type 2). They use the stochastic Moran model on a regular two-dimensional lattice as described in Foo et al. (2014). To estimate and compute the parameters for their model they use age-specific incidence rates from the Surveillance, Epidemiology, and End Results (SEER) program of the National Cancer Institute (18 registries, 2000–2012) in a Bayesian framework. Ryser et al. (2016) computed the survival function, the probability density function of the local field radius, and the probability of harboring at least two clonally unrelated fields in the head and neck region with respect to the mean age at smoking initiation to diagnosis with invasive cancer. They found that there is a strong dependence of the local field size on age at diagnosis, with a doubling of the expected field diameter between ages at diagnosis of 50 and 90 years. Further the probability of harboring multiple clonally unrelated fields at the time of diagnosis were found to increase substantially with patient age. As a result of these discoveries they suggest that patient age at diagnosis is a critical predictor of the size and multiplicity of precancerous lesions.

Our extensions of the model of Foo et al. (2014) will be in the framework of a hybrid cellular automaton as developed by Gerlee and Anderson (2007). Gerlee and Anderson (2007) created a hybrid cellular automaton to model the effect of various micro-environmental factors on solid tumor growth. Their model is a hybrid cellular automaton because the rule of the automaton depends upon the output of a neural network and partial differential equations. The cellular automaton is comprised of two cell types: an empty cell (normal cell) and a tumor cell. The neural network is used to approximate the relationship between the micro-environmental variables and the phenotype of a cell. The partial differential equations are used to model the spread of the various micro-environmental variables in the domain of consideration. While Gerlee and Anderson (2007) considered tumor growth, they did not specifically study the cancer field effect.

For the neural network they use a multi-layer perceptron (MLP), with input being the output of the partial differential equation for the cell at a location (*x, y*) and output being a vector of likelihoods of a phenotype and movement occurring at a time-step. The hidden layer of the MLP represents the genes and hence the neural network attempts to replicate the genotype-phenotype relationship. They consider the phenotypes proliferation (P), quiescence (Q), and apoptosis (A). Each time-step represents a cell cycle so that a single phenotypic action will occur once per time step for each cell. The maximum of the likelihoods between P, Q, and A determines which phenotypic action occurs. If the likelihood of movement is sufficiently large then the cell is allowed to move. The quiescent state is used to describe any normal activity of the cell which is not one of the three actions explicitly modeled above.

We extend Gerlee's model by (i) considering six cell types (normal tissue cells NTC, mutated normal tissue cells MNTC, normal stem cells NSC, mutated normal stem cells MNSC, cancer stem cells CSC, tumor cells TC, and empty cells) (ii) including transit amplifying cells (iv) including four phenotypic actions (proliferation, quiescence, apoptosis, differentiation) (v) including 10 gene expression levels for TP53, TP73, RB, TP21, T16, EGFR, CCDN1, MYC, PIK3CA, and RAS. (vi) following cell lineages, and (vii) considering full or partial surgical removal of cancer and cancer field cells.

## 2. The hybrid cellular automaton model

Here, we develop a hybrid cellular automaton (CA) model for the cancer field effect. We model on three distinct levels, a model for the carcinogen distribution, a neural network for the gene expression and a model for the cell dynamics over time.

We use carcinogen concentration function *c*_*i*_(*x, y*) to describe the carcinogen input on a two dimensional domain, where the index *i* is used to distinguish different carcinogens, for example *i* = 1 for smoking induced carcinogens, and *i* = 2 for alcohol induced carcinogens. In our simulations we consider a wide variety of carcinogen distributions *c*_*i*_(*x, y*), including a uniform, smooth spatial patterns, and random distributions. We found that the Gaussian distribution described below gave the most instructive results, hence we only report those. We choose a Gaussians centered in the domain middle as


(1) $$\begin{array}{l} c_{i}\left(x,y\right)=\exp\left(-\frac{1}{2}\frac{{\left(x-\mu \right)}^{2}+{\left(y-\mu \right)}^{2}}{\sigma^{2}}\right),\;\mu =\frac{N}{2}-1,\;\sigma =\frac{N}{15}; \end{array}$$

where *N* is the domain size (number of grid cells), assuming a square domain is used.

### 2.1. Gene expression neural network

**Figure 1:**
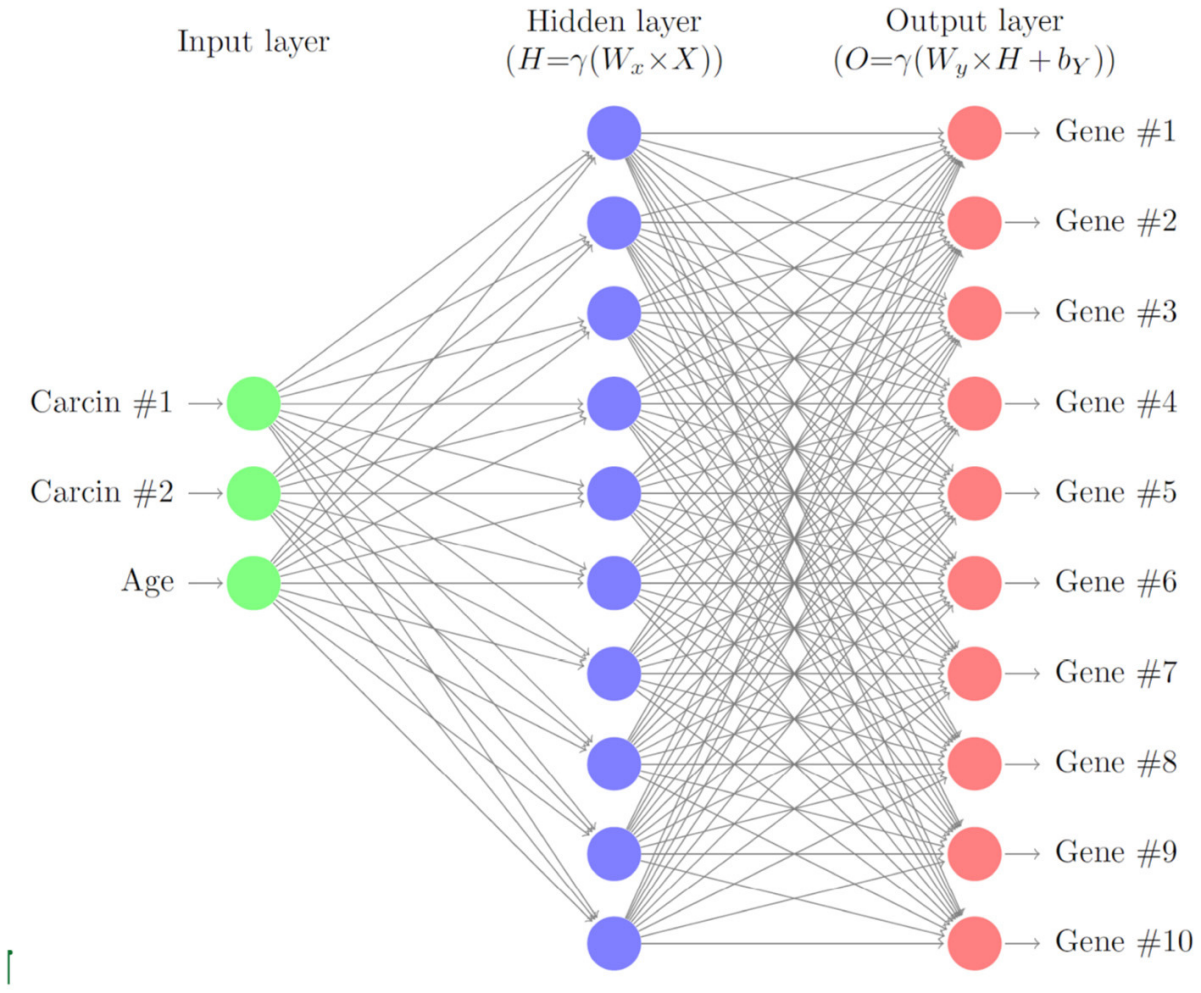
Schematic of the gene neural network. As input we use the carcinogen concentrations and the cell age. The hidden layer relates the carcinogens to the gene expression levels, which are the output of the neural network.

In this section, we describe a deep neural network with one hidden layer to account for varying levels of gene expressions through cell age and carcinogenic onslaught. A schematic of the layers of the neural network is given in Figure 1. We consider *G*∈ℕ genes that are biomarkers to the considered cancer type. Later we will model the gene expressions for HNSCC of the 10 genes TP53, TP73, RB, TP21, TP16, EGFR, CCDN1, MYC, PIK3CA, and RAS. Here, we formulate the model in general terms first.

#### 2.1.1. General case

A good general reference for neural networks can be found in Hastie et al. (2009). The gene expression of each gene is represented by the function


(2) $$\begin{array}{l} e_{j}\left(t\right)\in \mathbb{R},\;j=1,2,...,G. \end{array}$$

The gene expression is a non-dimensional value that is zero when the expression is normal, negative when it is under-expressed, and positive when it is over-expressed. The gene expression of each gene changes over time based upon a simple multi-layer perceptron (MLP). The input of the MLP is the vector


(3) $$\begin{array}{l} X\left(t\right):={\left[{\left\{c_{i}\left(t-1\right)\right\}}_{i=1,...,C},\alpha \left(t-1\right)\right]}^{T}\in {\mathbb{R}}_{+}^{C+1}, \end{array}$$

where *c*_*i*_(*t*) are the carcinogen concentrations, *C* denotes the number of carcinogen considered and α(*t*) is the age of the cell. The cell age α is measured for each cell after mitosis. Changes in gene expression are based upon the carcinogens in the environment of the cell and the age, which essentially means we are looking at the effects of the carcinogens and replication errors as a cell ages. The output of the MLP is given by


(4) $$\begin{array}{l} Y\left(t\right):={\left[{\left\{{\bar{\delta }}_{j}\left(t\right)\right\}}_{j=1,2,...,G}\right]}^{T}\in {\mathbb{R}}^{G}, \end{array}$$

where ${\bar{\delta }}_{j}\left(t\right)$ is the computed maximum possible change in gene expression for gene *j*. The amount the gene *j* will be mutated in a time-step is a random sample from the uniform distribution multiplied by ${\bar{\delta }}_{j}\left(t\right)$.

***Y***(*t*) is computed using matrix multiplication, addition and application of a non-linear transform. The hidden layer is computed by


(5) $$\begin{array}{l} H\left(t\right):=\gamma \left(W_{X}X\left(t\right)\right)\in {\mathbb{R}}^{G}, \end{array}$$

where


(6) $$\begin{array}{l} \gamma \left(\xi \right):=\frac{\xi }{\sqrt{1+\nu \xi^{2}}},\in \left(\frac{-1}{\sqrt{\nu }},\frac{1}{\sqrt{\nu }}\right) \end{array}$$

is the non-linear activation function that is applied element wise to a vector, and $W_{X}\in {\mathbb{R}}^{G\times C+1}$ is a weight matrix. Note that the activation function is chosen to ensure $|{\bar{\delta }}_{j}\left(t\right)|<\frac{1}{\sqrt{\nu }}$, hence allowing us to control the maximum amount the expression of gene *j* can change in a time-step via ν. After the hidden layer is computed the output is computed by


(7) $$\begin{array}{l} Y\left(t\right)=\gamma \left(W_{Y}H\left(t\right)+b_{Y}\left(t\right)\right), \end{array}$$

where $W_{Y}\in {\mathbb{R}}^{G\times G}$ is a weight matrix and $b_{Y}\left(t\right)\in {\mathbb{R}}^{G}$ is a bias vector.

Biologically speaking $W_{X}^{\left(i,j\right)},i\in \left[1,G\right],j\in \left[1,C\right]$ represents how carcinogen *i* influences gene *j*, $W_{X}^{\left(i,C+1\right)}$ represents whether cell age influences gene *i*, $W_{Y}^{\left(i,j\right)}$ represents whether gene *i* influences gene *j*, and $b_{Y}^{\left(i\right)}\left(t\right)$ denotes whether gene *i* has a higher or lower chance of gene expression changes relative to other genes. Note that if a value in the weight matrices is negative it means there is a negative relationship, if it is positive it means there is a positive relationship, and finally if it is zero it means there is no relationship.

We assume that as the cell ages, replication errors increase in frequency and cause random changes in gene expression. In the case of determining how age affects each gene, the values of $W_{X}^{\left(i,C+1\right)},i\in \left[1,G\right]$ are randomly made positive or negative at every time-step by sampling a Bernoulli random variable *b*_*i, t*_, with success probability parameter *p* = 0.5, and scaling $W_{X}^{\left(i,C+1\right)},i\in \left[1,G\right]$ by ${\tilde{\alpha }}_{i,t}\in \left\{\pm 1\right\}$ where


(8) $$\begin{array}{ll} b_{i,t} & \sim \text{Bernoulli}\left(p=0.5\right) \end{array}$$

(9) $$\begin{array}{ll} {\tilde{\alpha }}_{i,t} & =2b_{i,t}-1. \end{array}$$

Let *z*_*j*_~Uniform(0, 1), *j* = 1, …, *G*, be independent uniform random variables. The gene expression, *e*_*j*_(*t*), of a gene is updated by


(10) $$\begin{array}{l} e_{j}\left(t\right)=e_{j}\left(t-1\right)+z_{j}{\bar{\delta }}_{j}\left(t\right). \end{array}$$

A gene *j* is considered to be mutated if its gene expression is above the threshold value $\bar{M}\in {\mathbb{R}}_{+}$, i.e., $|e_{j}\left(t\right)|\geq \bar{M}$. The bias for a gene *j*, $b_{Y}^{\left(j\right)}\left(t\right)$, is updated through the relation


(11) $$b_{Y}^{\left(j\right)}(t)=\{\begin{array}{ll} \beta , & e_{j}(t-1)\geq \bar{M}\\ -\beta , & e_{j}(t-1)\leq -\bar{M}\\ 0, & \text{otherwise} \end{array},$$

where β∈ℝ_+_ is preset parameter.

#### 2.1.2. Neural network parameters for the HNSCC case

For the case of HNSCC we make the following choices. We consider the *G* = 10 genes TP53, TP73, RB, TP21, TP16, EGFR, CCDN1, MYC, PIK3CA, and RAS. The type of gene is summarized in Table 1.

**Table 1:** List of 10 genes that are relevant for HNSCC.

**Index**	**Gene**	**Gene-type**	**Regulation**	**Phenotypes**	**Carcinogen 1**	**Carcinogen 2**
1	TP53	Tumor-suppressor	Down	↑: *p*	Up	Down
				↓: *a*, *q*		
2	TP73	Tumor-suppressor	Down	↓: *a*		
3	RB	Tumor-suppressor	Down	↑: *p*, *d*		Down
				↓: *q*		
4	TP21	Tumor-suppressor	Down	↑: *p*	Up	Down
5	TP16	Tumor-suppressor	Down	↑: *p*	Up	
6	EGFR	Oncogene	Up	↑: *p*	Up	Up
7	CCDN1	Oncogene	Up	↓: *a*	Up	Up
8	MYC	Oncogene	Up	↑: *p*, *d*		Up
				↓: *a*		
9	PIK3CA	Oncogene	Up	↓: *a*		Up
10	RAS	Oncogene	Up	↑: *p*, *d*	Up	Up
				↓: *a*		

We indicate the type of gene as tumor suppressor or oncogene and show how the phenotypes change as the gene is up-or down regulated. The abbreviations *p, a, q, d* stand for the phenotypic actions of proliferation, apoptosis, quiescence, and differentiation, respectively. The column “regulation” indicates the expression level changes that make the cell more cancerous. The column “Phenotypes” indicates the relative change in *p, a, q, d*. In the last two columns we list how the given carcinogen changes the corresponding gene expression. Carcinogen 1 and 2 reflect the typical effects of smoking and alcohol consumption, respectively.

The weight matrix associated with the input of the neural network (5) is given by:


(12) $$\begin{array}{l} W_{X}\left(t\right)=\left[\begin{array}{ccc} 1 & -1 & {\tilde{\alpha }}_{1,t}10^{-7}\\ 0 & 0 & {\tilde{\alpha }}_{2,t}10^{-7}\\ 0 & -1 & {\tilde{\alpha }}_{3,t}10^{-7}\\ 1 & -1 & {\tilde{\alpha }}_{4,t}10^{-7}\\ 1 & 0 & {\tilde{\alpha }}_{5,t}10^{-7}\\ 1 & 1 & {\tilde{\alpha }}_{6,t}10^{-7}\\ 1 & 1 & {\tilde{\alpha }}_{7,t}10^{-7}\\ 0 & 1 & {\tilde{\alpha }}_{8,t}10^{-7}\\ 0 & 1 & {\tilde{\alpha }}_{9,t}10^{-7}\\ 1 & 1 & {\tilde{\alpha }}_{10,t}10^{-7} \end{array}\right], \end{array}$$

where ${\tilde{\alpha }}_{i,t}$ is defined by (9). As sufficient data was unavailable we assumed that each carcinogen has a weight of 1, −1, or 0 for each gene depending on how the carcinogen effects that gene. For example since ethanol tends to upregulate TP53 then $W_{X}^{11}=1$. The impact of alcohol and smoking induced carcinogens on gene mutations was taken from the large online data bases (PubChem, 2023a,b), and are listed in the last two columns in Table 1.

We assume that each gene has the same mutation rate which causes the last column in *W*_*X*_, that is associated with mutations caused by transcription errors due to cell age, to have one value. The mutation rate was chosen based upon the human genomic mutation rate being approximately 2.5 × 10^−8^ per base per generation (Nachman and Crowell, 2000). The weight matrix associated with the output of the neural network (7) is given by:


(13) $$\begin{array}{l} W_{Y}=\left[\begin{array}{cccccccccc} 1.00 & 0 & 0.01 & 0 & 0 & 0 & 0 & 0 & 0 & 0\\ 0.01 & 0.1 & 0 & 0 & 0 & 0 & 0 & 0 & 0 & 0\\ 0.01 & 0 & 0.3 & 0 & 0 & 0 & 0 & 0 & 0 & 0\\ 0.01 & 0 & 0 & 0.1 & 0 & 0 & 0.01 & -0.01 & 0 & 0\\ 0.01 & 0 & 0 & 0 & 0.1 & 0 & 0 & 0 & 0 & 0\\ 0.01 & 0 & 0 & 0 & 0 & 0.1 & 0 & 0 & 0 & 0\\ 0.01 & 0 & 0.01 & 0 & 0 & 0 & 0.2 & 0 & 0 & 0.01\\ 0.01 & 0 & 0 & 0 & 0 & 0 & 0 & 0.3 & 0 & 0.01\\ 0.01 & 0 & 0 & 0 & 0 & 0 & 0 & 0 & 0.1 & 0\\ 0.01 & 0 & 0 & 0 & 0 & 0 & 0 & 0.01 & 0 & 0.3 \end{array}\right]. \end{array}$$

The main diagonal of the above matrix gives the main weights for each gene with $W_{Y}^{11}$ being the highest as it is TP53. Each diagonal value was given a default of 0.1 and it is increased by 0.1 for each gene it calls or is related to, so TP53 gets a value of 1 because it is assumed all the genes relate to TP53. Each column describes the relations between the other genes and the gene associated with the main diagonal value of that column, where if the gene is upregulated by the diagonal gene it gets a value of 0.01 and when it downregulates the gene it gets a value of −0.01. The magnitude of the values in the matrix were chosen by trial and error since there is not sufficient data to complete the matrix with accurate values.

The activation function (6) parameter is given by ν = 10^6^. The value of ν results in the neural network outputting values in the range $\left(\frac{-1}{\sqrt{\nu }},\frac{1}{\sqrt{\nu }}\right)=\left(-10^{-3},10^{-3}\right)$ and was chosen so to keep the maximum amount each gene can change to a reasonable figure.

Finally the mutation bias vector update function (11) parameter is given by β = 10^−3^. The value of β was chosen to correspond with the maximum output value of the neural network, so that when a gene is mutated, the neural network will always output the maximum value.

### 2.2. Cellular automaton

We consider a two dimensional regular grid with *N* grid cells. Each cell can be occupied by a cell, or be empty. The six cell classes that we consider are normal tissue cells (NTC, brown), mutated normal tissue cells (MNTC, green), normal stem cells (NSC, blue), mutated normal stem cells (MNSC, yellow), cancer stem cells (CSC, purple), and tumor cells (TC, red), plus an empty cell (white) (see Table 2).

**Table 2:** CA cell classes.

**Cell class**	***s(t)***	**Color**
Normal tissue cell (NTC)	0	**brown**
Mutated normal tissue cell (MNTC)	1	**green**
Normal stem cell (NSC)	2	**blue**
Mutated normal stem cell (MNSC)	3	**yellow**
Cancer stem cell (CSC)	4	**purple**
Tumor cell (TC)	5	**red**
Empty cell	6	**white**

The cell class in the CA is represented by *s*(*t*)∈{0, 1, ..., 6} with 0 = NTC, 1 = MNTC, 2 = NSC, 3 = MNSC, 4 = CSC, 5 = TC, 6 = empty.

Evolution of the model occurs in the following basic steps:

1. Given a carcinogen exposure, changes in gene expressions of a cell are computed by the neural network causing gene mutations to occur.
2. Based on the genetic profile, the cell executes one of four phenotypic actions: proliferation, quiescence, apoptosis, differentiation. If empty space is available in the neighborhood, the cell moves with a certain probability.

Here, we give a shortened description of the rules of the cellular automaton. A full description, involving mathematical formulas for all possible transitions, is given in the Appendix 1 (Supplementary material).

Each cell in the CA tracks the gene expression of the *G* genes in a vector defined by


(14) $$\begin{array}{l} E\left(t\right)=\left[{\left\{e_{j}\left(t\right)\right\}}_{j=1,...,G}\right]. \end{array}$$

The phenotype of a cell is tracked by a vector that contains probabilities for each type of phenotypic action occurring in a given time-step and is defined by


(15) $$\begin{array}{l} P\left(t\right)=\left[p\left(t\right),q\left(t\right),a\left(t\right),d\left(t\right)\right], \end{array}$$

where *p*(*t*) represents proliferation, *q*(*t*) represents quiescence, *a*(*t*) represents apoptosis, and *d*(*t*) represents differentiation. The probabilities are set such that ***P***(*t*) generates a probability distribution, so that


(16) $$\begin{array}{l} \overset{4}{\underset{i=1}{\sum }}P_{i}\left(t\right)\equiv p\left(t\right)+q\left(t\right)+a\left(t\right)+d\left(t\right)=1\text{and}P_{i}\left(t\right)\geq 0,\forall t. \end{array}$$

At a time-step in the CA a phenotypic action is chosen to occur by sampling from the probability distribution generated from ***P***(*t*). Since we do not want a cell to reproduce more than once in a time-step, each time-step represents the length of a typical cell cycle for the type of tissue under consideration.

When a NSC, MNSC, or CSC differentiate the resultant cell initially is a transit amplifying cell (TAC) for a set number of generations, Θ, after which it turns respectively into a NTC, MNTC, or TC. As a result of this each cell has two parameters $\bar{\tau }\left(t\right)\in \left\{0,1\right\}$ and $\bar{n}\left(t\right)\in \left\{0,...,\Theta \right\}$, where $\bar{\tau }\left(t\right)$ is a binary parameter used to determine if a cell is currently a TAC or not and $\bar{n}\left(t\right)$ is the number of generations a TAC cell lineage has produced. The parameters $\bar{\tau }\left(t\right)$ and $\bar{n}\left(t\right)$ are copied from parent to child cell and once $\bar{n}\left(t\right)=\Theta$ then $\bar{\tau }\left(t+1\right)=0$, $\bar{n}\left(t+1\right)=0$.

The final aspect of the cell that is tracked and represented in the overall cell state is the age of the cell, α(*t*)∈ℕ. Hence, the complete state of a cell in the CA is given by the vector that contains cell type *s*, age α, gene expression *E*, phenotye vector *P*, TAC state $\bar{\tau }$, and TAC generation $\bar{n}$ as


(17) $$\begin{array}{l} S\left(t\right)=\left[s\left(t\right),\alpha \left(t\right),E\left(t\right),P\left(t\right),\bar{\tau }\left(t\right),\bar{n}\left(t\right)\right] \end{array}$$

Each cell has a neighborhood that contains itself, the cardinal directions around it, and the cells directly NE, SE, SW, and NW of the cell. In CA theory this is called the Moore neighborhood (Gray, 2003). The boundary conditions of the grid are standard periodic boundary conditions for convenience. Other boundary conditions could be easily implemented too.

#### 2.2.1. Cell mutations

The chosen *G* genes are known genes related to the type of cancer being studied. For the case of HNSCC, (see Table 1). We define the vector ***T***∈{0, 1}^*G*^, where *T*_*j*_ = 0 represents a tumor suppressor gene and *T*_*j*_ = 1 represents an oncogene. A gene *j* is positively mutated toward cancer (positively mutated) if either it is a tumor suppressor gene and its gene expression is downregulated, $e_{j}\left(t\right)\leq -\bar{M}$, or it is an oncogene and its gene expression is upregulated, $e_{j}\left(t\right)\geq \bar{M}$, where $\bar{M}$ is the given threshold. At each time-step the gene expression of each gene is updated from the results of the gene expression neural network from Section 2.1. The changes in the gene expression allow the gene to become mutated or even go from mutated to non mutated (normally expressed).

We also consider that a mutated gene can influence another gene, where we assume that a positively mutated gene will cause a positive mutation of a related gene. A non-positively mutated gene will cause a negative mutation (mutation that regulates a gene toward normal expression) of a related gene. We express this interdependence through a matrix *R*∈{0, 1}^*G*×*G*^, where each entry, *R*_*ij*_, represents whether gene *i* is related to gene *j* with 0 = unrelated and 1 = related. Note that the matrix *R* is not necessarily symmetric as a gene *i* might regulate gene *j* but not vice versa.

We show in the Appendix 1 (Supplementary material) how this update is done mathematically.

#### 2.2.2. Update rules for phenotypic action

When a gene is mutated it can modify the probability of a phenotypic action occurring, *P*_*i*_(*t*). Hence the phenotypic actions *P*_*i*_ need to be updated for each cell at each time step. We define the matrix $\bar{U}\in {\mathbb{R}}^{4\times G}$, where each entry, ${\bar{U}}_{ij}$, is an increment to the probability of phenotypic action *i*, *P*_*i*_(*t*), under the circumstance that gene *j* is mutated and its' expression is upregulated. Similarly, we define the matrix $\bar{D}\in {\mathbb{R}}^{4\times G}$, where each entry, ${\bar{D}}_{ij}$, is an increment to the probability of phenotype action *i*, *P*_*i*_(*t*), under the circumstance that gene *j* is mutated and its' expression is downregulated. We define detailed rules for updates to the phenotypic action probabilities in Appendix 1 (Supplementary material). Note that we often choose $\bar{U}=-\bar{D}$ for symmetry. The sum of the phenotype vector equaling one is maintained by balancing the probability of each phenotype action against the probability of quiescence and quiescence equally against all the other phenotypic actions (see Appendix 1 in Supplementary material for more details).

#### 2.2.3. Update rules for cell class

A cell is considered mutated if it has more than or equal to Υ∈ℕ positively mutated genes. If a transition from a non-mutated cell to a mutated cell occurs, the phenotype vector is updated as described in Appendix 1 (Supplementary material).

Dedifferentiation is the process of a specialized cell reverting back to a non-specialized cell. In our model this is accomplished by a non stem cell becoming a stem cell. Dedifferentiation depends on a complex interaction of positive and negative feedback mechanisms involved in cell proliferation and gene expression. A recent review (Hillen and Shyntar, 2023) (and the references therein) discuss detailed modeling of these aspects. Here, dedifferentiation is used to help maintain the proper ratio of stem cells to non stem cells in the grid by dedifferentiating whenever the number of stem cells in the neighborhood of a non stem cell is less than or equal to some chosen value, Ŝ, or if the number of empty cells in the neighborhood of a non stem cell is less than or equal to some chosen value, Ê. To help reduce the number of cells dedifferentiating, the process is completed only when a random sample from the uniform distribution is less than or equal to some threshold, $\hat{D}\in {\mathbb{R}}_{+}\left(0,1\right)$.

We associate a fitness value to each cell, so that the cells can compete and the population contains only the healthiest, or in the case of mutated cells, the most positively mutated cells. The characteristics that affect the fitness are based upon work by Bowling et al. (2019). High fitness is characterized by a low apoptotic rate, a high proliferation rate, young age. Also cancer cells are considered to have a high fitness.

In addition to the four phenotypic actions, quiescent cells can move with a specified probability into a neighboring cell. A CSC or TC can move into an occupied neighboring cells, killing the occupant in the process. In this case, the probability of moving into an occupied cell is lower than the probability of moving into an empty cell.

CSCs and TCs are the only class of cells that can kill other cells when moving during quiescence. If the parent cell is a CSC or TC and the chosen cell has a higher fitness then the phenotypic action is accomplished only if a sample from some random variable is less than a threshold to kill, κ∈ℝ_+_(0, 1). A CSC can kill a TC and TC a CSC only if the fitness is lower.

At each time step, each non-empty cell in the CA grid chooses an action to execute and attempts to complete such action. Consider that the cell that is performing the action is located at ***x***^(*p*)^∈Ω. In certain cases the action will be performed upon a (randomly chosen) neighboring target cell, located at ***x***^(*c*)^∈Ω. As mentioned above, the target cell need not be empty. In the case of proliferation, and differentiation, the action can take place when the occupant of target cell has a lower fitness.

The lineage of each cell is tracked for the purpose of following tumor cell lineages from their origin, checking how many independent tumor masses form throughout the simulation, and whether the origin is monoclonal or polyclonal.

#### 2.2.4. CA model timeline

Each time-step has the following order of actions (1) Run the gene expression neural network, (2) Update the gene expressions based upon the output of the neural network in step 2, (3) Update the gene expressions via the gene instability process, (4) Update the phenotype vector based upon the gene expressions of each gene, (5) Update the states of each cell using the state transition process, (6) Apply the dedifferentiation process, (7) Apply the phenotypic action chosen by the cell for that time-step, and apply possible random walk to a neighboring empty cell, (8) Possibly perform tumor excision.

#### 2.2.5. CA parameters for HNSCC

The initial seed is set such that the domain has the following breakdown of each cell type: 64.5% normal tissue cells (NTC; brown), 6.5% normal stem cells (NSC; yellow), and 29% empty cells (white). The maximum number of TAC generations is given by Θ = 2. The chance a cell moves when it is quiescent is 0.25. The chance a tumor cell (TC; red) or cancer stem cell (CSC; purple) randomly kills another cell during movement, proliferation, or differentiation is 0.2. The chance that an SC or MSC becomes a CSC is 2.5 × 10^−6^. The chance a non stem cell becomes a stem cell through dedifferentiation is 10^−4^. If either there are no stem cells or there are at least six empty cells in the neighborhood of a non stem cell, then the process of dedifferentiation will be attempted. When an excision is performed the number of neighbors around a TC removed is two.

We consider 10 genes which are given in Table 1. We set the mutation threshold to $\bar{M}=0.1$ and the minimum number of positively mutated genes for a cell to be considered mutated to be four (Anandakrishnan et al., 2019). Using the last two columns of Table 1 and assuming each phenotypic action is modified at the same magnitude we obtain the phenotypic action increment matrices (see Section 2.2.2 and Appendix 1 in Supplementary material) given by:


(18) $$\begin{array}{l} \bar{D}=\left[\begin{array}{cccc} 10^{\text{-}6} & -10^{\text{-}6} & -10^{\text{-}6} & 0\\ 0 & 0 & -10^{\text{-}6} & 0\\ 10^{\text{-}6} & -10^{\text{-}6} & 0 & 10^{\text{-}6}\\ 10^{\text{-}6} & 0 & 0 & 0\\ 10^{\text{-}6} & 0 & 0 & 0\\ -10^{\text{-}6} & 0 & 0 & 0\\ 0 & 0 & 10^{\text{-}6} & 0\\ -10^{\text{-}6} & 0 & 10^{\text{-}6} & -10^{\text{-}6}\\ 0 & 0 & 10^{\text{-}6} & 0\\ -10^{\text{-}6} & 0 & 10^{\text{-}6} & -10^{\text{-}6} \end{array}\right],\;\bar{U}=-\bar{D}. \end{array}$$

Using Table 1, we can create the gene type vector, ***T***, that is used in (A6), (A7), and (A28) which is given by:


(19) $$\begin{array}{l} T={\left[\begin{array}{cccccccccc} 0 & 0 & 0 & 0 & 0 & 1 & 1 & 1 & 1 & 1 \end{array}\right]}^{T}, \end{array}$$

where 0 means the gene is a tumor-suppressor and 1 means it is an oncogene.

Using Table 3, we can create the gene relationship matrix, *R*, that is used in (A7) which is given by:


(20) $$\begin{array}{l} R=\left[\begin{array}{cccccccccc} 0 & 0 & 1 & 0 & 0 & 0 & 0 & 0 & 0 & 0\\ 1 & 0 & 0 & 0 & 0 & 0 & 0 & 0 & 0 & 0\\ 1 & 0 & 0 & 0 & 0 & 0 & 0 & 0 & 0 & 0\\ 1 & 0 & 0 & 0 & 0 & 0 & 1 & 1 & 0 & 0\\ 1 & 0 & 0 & 0 & 0 & 0 & 0 & 0 & 0 & 0\\ 1 & 0 & 0 & 0 & 0 & 0 & 0 & 0 & 0 & 0\\ 1 & 0 & 1 & 0 & 0 & 0 & 0 & 0 & 0 & 1\\ 1 & 0 & 0 & 0 & 0 & 0 & 0 & 0 & 0 & 1\\ 1 & 0 & 0 & 0 & 0 & 0 & 0 & 0 & 0 & 0\\ 1 & 0 & 0 & 0 & 0 & 0 & 0 & 1 & 0 & 0 \end{array}\right], \end{array}$$

where 0 means the genes are not related and 1 means the genes are related. Note that in the above matrix we assumed that TP53 is related to all the genes. The main diagonal is zero so that genes cannot modify themselves during the genetic instability phase of the model. The chance that a gene modifies the gene expression of another or that the body tries to repair the gene over or under expression is 0.45. The maximum amount a gene expression can be changed during the gene instability stage is $\frac{1}{\sqrt{\nu }}$. We let ${\bar{a}}_{1}=\frac{\tilde{c}}{{\bar{c}}_{1}}$ and ${\bar{a}}_{2}=\frac{\tilde{c}}{{\bar{c}}_{2}}$ be the initial probabilities of apoptosis for a normal tissue cell and normal stem cell. Where $\tilde{c}$ is the length of the cell cycle in hours, ${\bar{c}}_{1}$ is the life span of a cell, and ${\bar{c}}_{2}$ is the life span of a stem cell. The initial phenotype matrix that is used in Equation (A25) is given by:


(21) $$\begin{array}{l} \tilde{P}=\left[\begin{array}{cccc} {\bar{p}}_{1}{\bar{a}}_{1} & {\bar{a}}_{1} & 1-{\bar{a}}_{1}\left({\bar{p}}_{1}+1\right) & 0\\ {\bar{p}}_{1}{\bar{a}}_{1} & \frac{{\bar{a}}_{1}}{\bar{\alpha }} & 1-{\bar{a}}_{1}\left({\bar{\alpha }}^{-1}+{\bar{p}}_{1}\right) & 0\\ {\bar{p}}_{2}{\bar{a}}_{2} & {\bar{a}}_{2} & 1-{\bar{a}}_{2}\left({\bar{p}}_{2}+1\right)-\bar{d}\tilde{d} & \bar{d}\tilde{d}\\ {\bar{p}}_{2}{\bar{a}}_{2} & \frac{{\bar{a}}_{2}}{\bar{\alpha }} & 1-{\bar{a}}_{2}\left({\bar{\alpha }}^{-1}+{\bar{p}}_{2}\right)-\bar{d}\tilde{d} & \bar{d}\tilde{d}\\ {\bar{p}}_{2}{\bar{a}}_{2} & \frac{{\bar{a}}_{2}}{5{\bar{\alpha }}^{2}} & 1-{\bar{a}}_{2}\left({\left(5\bar{\alpha }\right)}^{-2}+{\bar{p}}_{2}\right)-\bar{d}\tilde{d} & \bar{d}\tilde{d}\\ {\bar{p}}_{1}{\bar{a}}_{1} & \frac{{\bar{a}}_{1}}{5{\bar{\alpha }}^{2}} & 1-{\bar{a}}_{1}\left({\left(5\bar{\alpha }\right)}^{-2}+{\bar{p}}_{1}\right) & 0 \end{array}\right], \end{array}$$

where ${\bar{p}}_{1}$ is the proliferation factor for normal tissue cell types, ${\bar{p}}_{2}$ is the proliferation factor for normal stem cell types, $\bar{\alpha }$ is the apoptotic factor, $\bar{d}$ is the differentiation factor, and $\tilde{d}$ is the probability of differentiation occurring neglecting competition between cells. The cell cycle length can range anywhere between 8 and 24 h for the various cells in the body, since we are analyzing the tongue we will use $\tilde{c}=10\text{h}$ (Beidler and Smallman, 1965). The lifespan of a taste bud is 250 ± 50 h (Beidler and Smallman, 1965), so ${\bar{c}}_{1}=250\text{h}$. The lifespan of a typical stem cell is around 25, 550 h (Sieburg et al., 2011), so ${\bar{c}}_{2}=25550\text{h}$. We set $\bar{\alpha }=1.625$, ${\bar{p}}_{1}=0.65$, ${\bar{p}}_{2}=14.75$, and $\bar{d}=1.485$ so that equilibrium in the tissue is maintained when there are no carcinogens in the domain. Note that ${\bar{p}}_{1}$ is less than 1, since we want most of the new cells to come from TACs created by SCs, because, biologically speaking, normal tissue cells rarely proliferate. Since each TAC produces a certain number of generations, given by Θ, then it will produce 2^Θ+1^−2 new cells so we set


(22) $$\begin{array}{l} \tilde{d}=\frac{1}{2^{\Theta +1}-2}. \end{array}$$

When a cell is a TAC the probability of proliferation increases by $\frac{1}{3}$, so that it will create its Θ generations in as few time-steps as possible, assuming there is enough available space. The chance that a gene modifies the probability of a phenotypic action is given by 0.35. The maximum value a gene can modify the phenotypic action by is 10^−6^.

**Table 3:** Shows which genes are activated by certain genes.

**Gene**	**Gene activation's**
TP53	TP21, TP16, RB
RB	TP53, CCDN1
CCDN1	TP21
MYC	TP21 (de-activates), Ras
RAS	CCDN1, MYC

## 3. Results

In this section, we present the results of simulations of the CA model as applied to HNSCC. We will explore what impact the following have on the results: grid size, number of carcinogens, carcinogen concentration, and excising the tumor vs. excising the entire field. We will discuss field growth, changes in probabilities of phenotypic actions over time, mutation spread rates, and the number of lineages. By tracking lineages we will also check monoclonal vs. polyclonal origins.

### 3.1. Equilibrium

We first ensure that the model can maintain a healthy tissue structure, if not exposed to a carcinogen. This will show that in our base model random mutations alone cannot cause cancer on the time scales considered here due to the low mutational rate of genes in the healthy body, and the fact that the body is well adept at repairing mutations as they occur. We run the simulation on a 128 × 128 grid for 8,766 time-steps (about 120 months), and as stated above, with no carcinogens.

**Figure 2:**
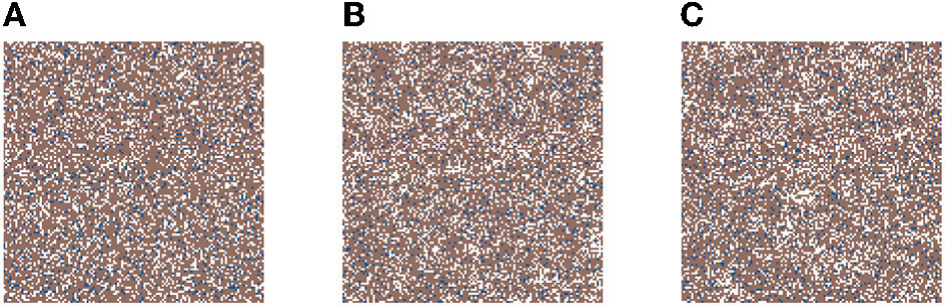
This figure includes three time-steps. The grid size is 128 × 128 and no active carcinogens are present. Using the color map for the cell classes as provided in Table 2. These show **(A)** the initial seed of the simulation *t* = 0, in **(B)** the domain (tissue) at the halfway point of the simulation *t* = 4, 383 steps = 5 years, and in **(C)** the final time-step at *t*= 10 years.

In Figure 2, we present three time-steps from a simulation where no carcinogens were included, with normal tissue cells (NTC) as the brown cells, normal stem cells (NSC) as the blue cells, and empty cells are white. Figure 2 shows (Figure 2A) the initial seed, Figure 2B the domain (tissue) at the halfway point of the simulation (5 years), and Figure 2C the final time-step (10 years). We observe that the tissue stayed in equilibrium. The changes through time are due to cell movement and the natural birth and death processes. The figures show that, as desired, no mutated cells (green, yellow) arise and thus no cancer stem cells (purple) or tumor cells (red) are formed.

### 3.2. Results with carcinogens

**Figure 3:**
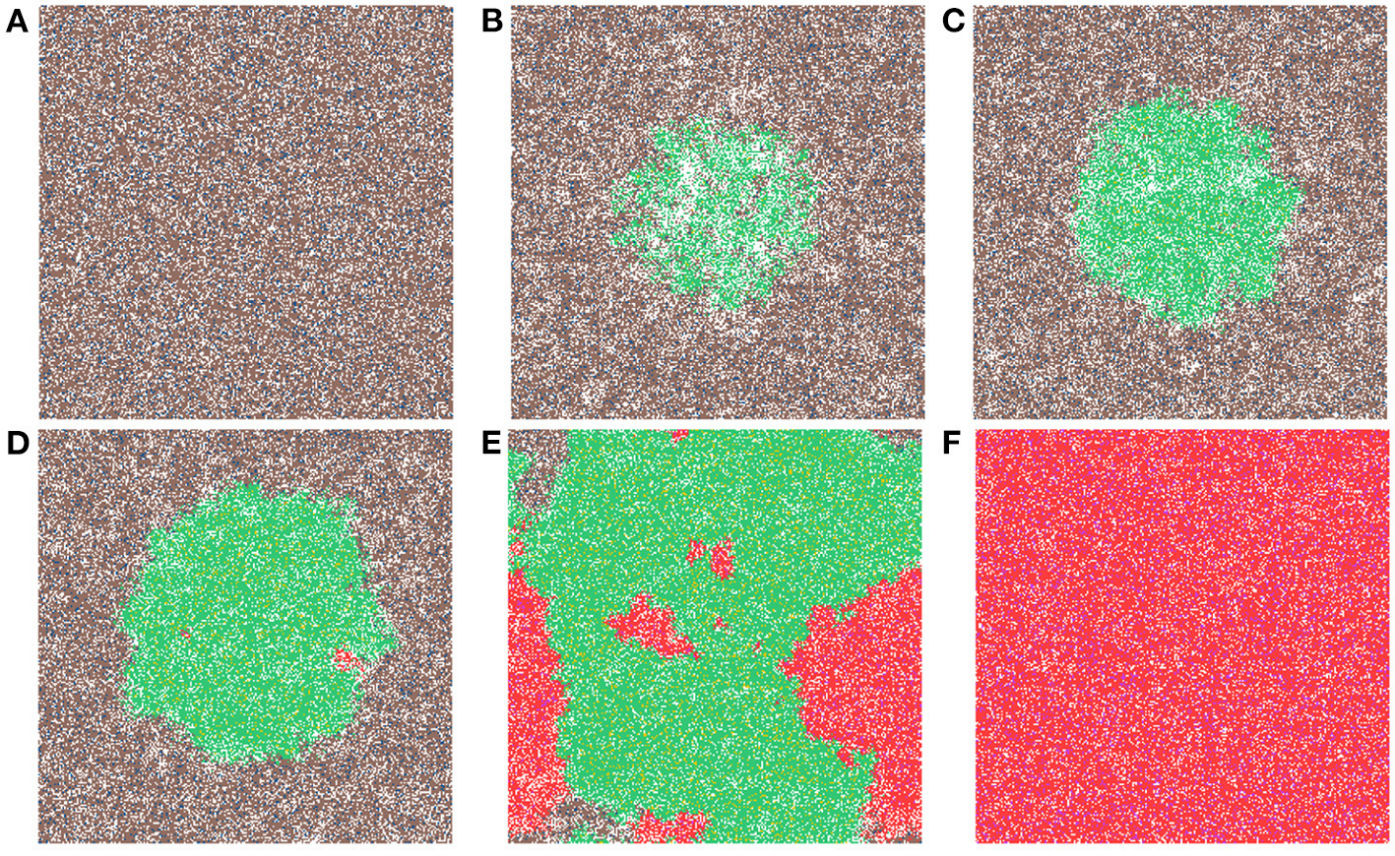
This figure includes time-steps illustrating the development of a cancer field and cancer cells using the color map for the cell classes as provided in Table 2. These show **(A)** the initial seed, **(B)** the cancer field at its early development, **(C)** the cancer field further developing but prior to cancer, **(D)** the first stages of cancer development, **(E)** further cancer growth, and **(F)** the final time-step. Parameters are as follows: grid size 256 × 256, carcinogen spatial distribution 2, both carcinogens activated. A video of this simulation can be found on youtube: https://youtu.be/eKxsrSoDiKs.

Now we study simulations where carcinogens are present and cause mutations and, ultimately, cancer. Figure 3 illustrates the development of a cancer field and tumors within it, where smoking and alcohol consumption are simulated using carcinogen spatial distribution (1). The various time-steps show (Figure 3A) the initial seed, Figure 3B the cancer field at its early development, Figure 3C the cancer field further developing prior to cancer, in Figures 3D–F the multiple stages of cancer development. The color map for the cell classes is as provided in Table 2. The cancer field (green) is initially minimal and undeveloped, but over time it evolves and matures, eventually forming tumors (red and purple). These tumors grow and outpace the growth rate of the cancer field.

We have uploaded four video files to youtube that show the dynamics of our hybrid CA model. Each video shows from left to right the carcinogen, the CA dynamics, and the 20 largest cell lineages. The grid size is 256 × 256 in all these simulations (see Table 4).

**Table 4:** List of youtube videos for the CA dynamics, where we show from left to right the carcinogen, the CA simulation, and the 20 largest cell lineages.

**Youtube video**	**Showing this effect**
**https://youtu.be/**	
eKxsrSoDiKs	General scenario
Gtf6MoxXCkM	Different carcinogen distribution
zngGzjSlPwU	Excision of cancer cells
EOFI4Ai1A9U	Excision of cancer and field

#### 3.2.1. Field development

Regardless of changes to parameters we observe that the field begins to form where the carcinogen is most concentrated; this can be verified with Figure 3B. Initially, the field is made up of only mutated normal tissue cells (green cells) and mutated normal stem cells (yellow cells). We defined the cancer field as the areas of the domain that contain cells from the mutated cell classes thus, the figure shows the cancer field growth over time. Typically, the first mutated cell is a MNTC due to there being a higher number of NTC compared to NSC. The field grows outwards as it takes over normal tissue. Once the first CSC arises, cancer grows quickly. We observe a number of focal regions of cancer development, and if untreated, it will fully overgrow the domain. The different focal points in Figure 3E correspond to different cell lineages, as we show later.

**Figure 4:**
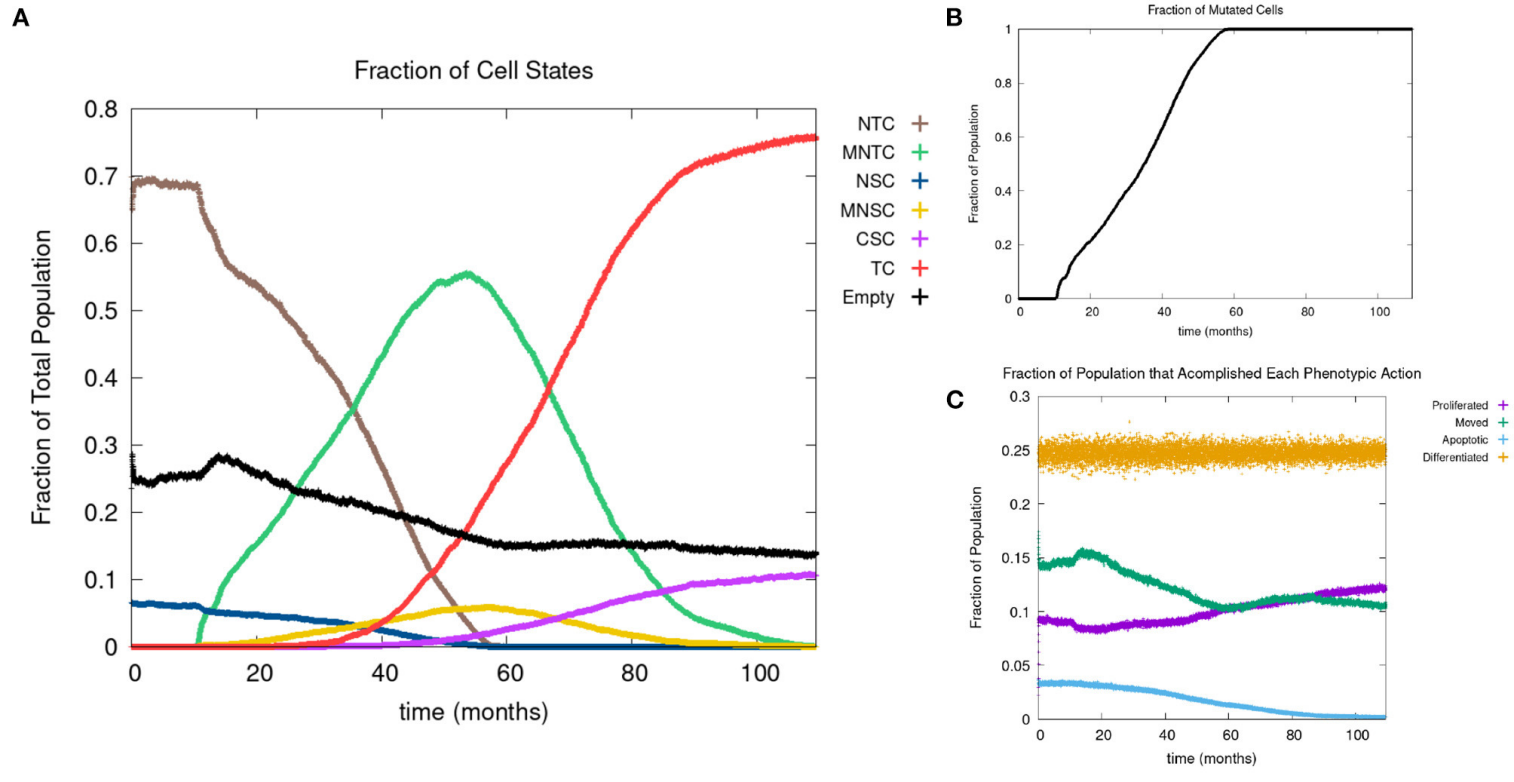
**(A)** Fraction of the various sub-populations as function of time. **(B)** Time course of the fraction of mutated cells, thus illustrating the cancer field growth over time. **(C)** Evolution of phenotypic probabilities of proliferation, apoptosis, quiescence, and differentiation. Parameters are as follows: grid size 256 × 256, carcinogen spatial distribution 2, both carcinogens activated shows the fraction of cancer cells over time. Parameters are as follows: grid size 256 × 256, carcinogen spatial distribution 2, both carcinogens activated.

In Figure 4A, we show the time evolution of the fraction of all cell classes. The cancer field begins to from once the green curve starts to grow. Once the field has developed and grown large enough, the odds of a NSC or MNSC becoming a CSC increases. Soon after the emergence of the first CSC, TCs begin to form and grow in a logistic fashion into the domain. As cancer grows the normal tissue (brown) and field tissue (green) is reduced.

#### 3.2.2. Mutational evolution

**Figure 5:**
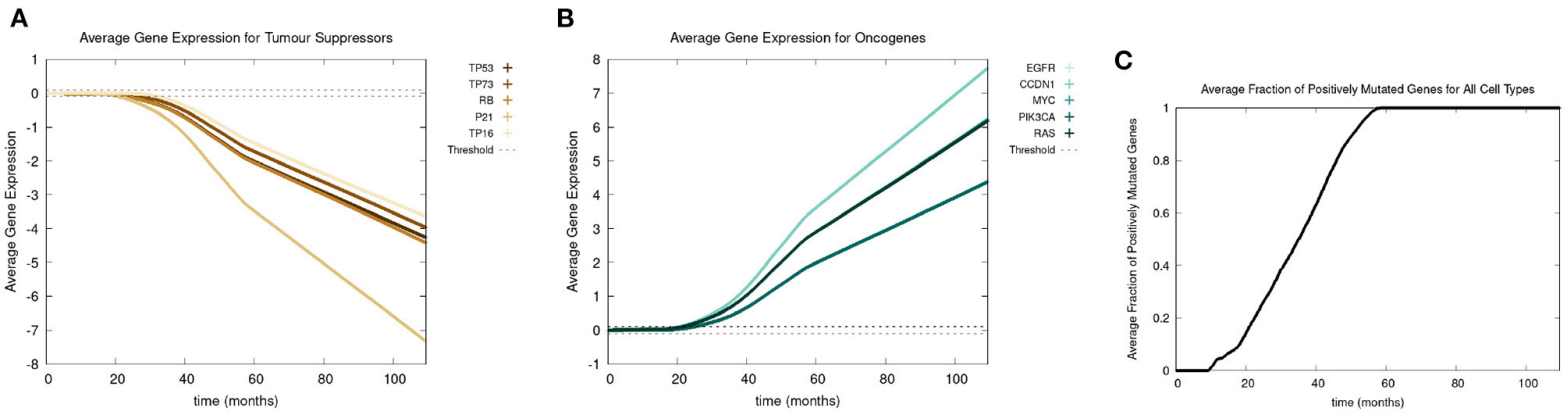
In this figure, we show the mutational evolution of the genes. The time course of the average gene expression are shown for in **(A)** the tumor suppressor genes, in **(B)** the oncogenes. In **(C)**, we show the time course of the fraction of genes that are positively mutated. Parameters were chosen as follows: grid size of 256 × 256, carcinogen spatial distribution 2 was used, both carcinogens were activated.

Recall we use the term “positive mutation” for mutations that promote cancer (i.e., upregulation of an oncogene or downregulation of a tumor suppressor gene). In Figure 5, we show various graphs that represent the mutational evolution of the genes over time. In Figures 5A, B the average gene expression is illustrated for first the tumor suppressors and secondly the oncogenes. In Figure 5C, we show the time evolution of the fraction of genes that are positively mutated. In Figure 5A all the tumor suppressor genes are downregulated, hence positively mutated. Other than RB, which decreases at a faster rate, the gene expressions of all the other tumor suppressor genes decrease at a similar rate. Figure 5B displays that the oncogenes are upregulated, therefore positively mutated. The gene expressions of the oncogenes increase at a similar rate except CCDN1 and RAS, which increase at a faster rate. We see that the gene expressions between the genes can vary significantly, principally with P21 and CCDN1. These two genes mutate because they are related to the most genes, and therefore have a higher weight in the MLP output weight matrix, *W*_*y*_, in Equation (13).

Figure 5C shows a lag of time before the first positively mutated genes occur, this is due to the low mutational rate. The initial spike in mutational rate at the onset of the first mutated cell is due to the relative size of the domain vs. the mutated cells. Once multiple genes become positively mutated the progression accelerates, due to changes in the expression of other genes caused by genetic instability, as displayed in the period starting at about 20 to 25 months. We observe that all the genes are positively mutated by about 25 months.

#### 3.2.3. Phenotypic evolution

Next we consider the evolution of a phenotypic actions of proliferation, quiescence, apoptosis, and differentiation as functions of time. Figure 4C illustrates the phenotypic evolution of these actions as the time evolution of the fraction of cells that underwent each phenotypic action at a given time step. The probability of apoptosis occurring decreases as the cell population moves toward being cancerous. This occurs since the majority of the genes become positively mutated causing apoptosis to decrease. While the probability of apoptosis decreases, the chance of proliferation and differentiation increases, which again is caused by positively mutated genes. Probability of differentiation increases at a slower rate than proliferation because fewer of the genes we consider influence differentiation. Finally, for the most part, the probability of quiescence remains stable—it goes slightly up and down, due to being balanced against the other phenotypic actions, and not many genes are influencing it, but otherwise it is at equilibrium. Figure 4 shows us that apoptosis and proliferation change the most over time, in particular, as apoptosis decreases, we see that proliferation increases, due to less cells dying before they can become more cancerous.

### 3.3. Grid size comparisons

**Figure 6:**
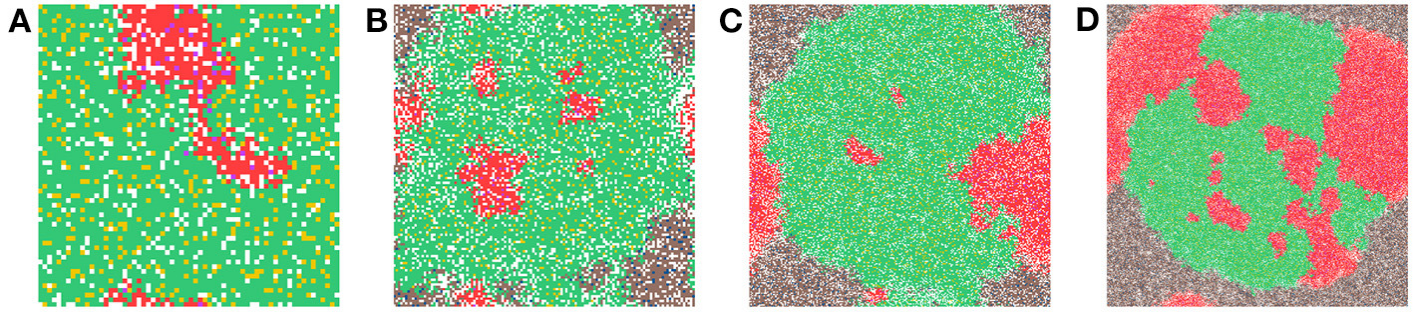
This figure shows a time-step from each of the grid sizes that were considered for comparison. In **(A)** the grid size is 64 × 64, in **(B)** the grid size is 128 × 128, in **(C)** the grid size is 256 × 256, and in **(D)** the grid size is 512 × 512. Parameters: both carcinogens were activated and carcinogen spatial distribution 2 was used.

In Figure 6, we show a sample time-step for each grid size we compare. In Figure 6A the grid size is 64 × 64, in Figure 6B the grid size is 128 × 128, in Figure 6C the grid size is 256 × 256, and in Figure 6D the grid size is 512 × 512. When comparing the grid sizes all the other parameters were the same, both carcinogens were activated and carcinogen spatial distribution (1) was used. We see that as the domain size increases, the tumor masses within it also increase.

The various grid sizes show slight differences in four ways, all predominantly due to the increase in the number of cells. Most of the events in the CA are probabilistic, as a result, almost automatically, as we increase the size of the grid, the chance of a probabilistic event increases as well. Therefore, the overall dynamics are the same for each grid size, but the timing of various main events differ slightly as will be illustrated with the following table.

In Table 5, we show the time-step at which the first mutated cell forms. The times are comparable and the first mutated cell is always an MNTC, due to the fact that a higher ratio of NTC than SC exists in the domain. We also compare the time-step the first CSC forms for each grid size. The variation between the smallest grid size as compared to the remaining three is substantial, due to the fact that the probability of a CSC forming is minuscule, attempting such an action within such a small grid size reduces the chances of these formations drastically in comparison to the larger grid sizes. We also note that the time for a first TC to develop from a CSC is short at about 3–4 time steps in all our simulations.

**Table 5:** In this table, we compare the time-step the first mutated cell forms, the time step where the first cancer stem cell (CSC) arises, and the number of cancer cell lineages at the end of the simulations, between the different grid sizes.

**Grid size**	**Time-step first mutated cell forms**	**Time step first CSC**	**# tumor cell lineages**
64 × 64	817	2,872	1
128 × 128	748	1,402	1
256 × 256	744	1,650	6
512 × 512	735	1,082	24

A notable difference arises if we count the cell lineages inside the cancerous tissue, since a larger domain simply has more space for different cell lineages to thrive. Table 5 shows the number of tumor cell lineages at the end of the simulation for each grid size.

### 3.4. Various carcinogen schedules

In this section, we consider the carcinogens from alcohol and smoking individually and in combination with one another. We consider several administration schedules for heavy and light smokers and drinkers.

**Figure 7:**
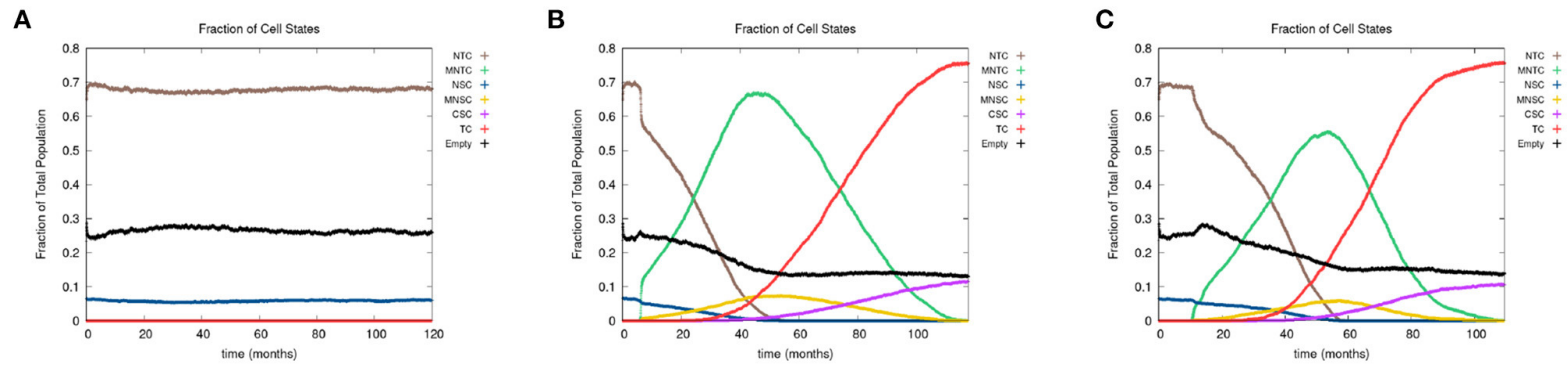
We show the time course of the fraction of cells in the different cell classes NTC, MNTC, NSC, MNSC, CSC, TC, and empty for **(A)** alcohol alone, **(B)** smoking alone, and **(C)** alcohol and smoking together. Grid size 256 × 256.

In Figure 7, we present the time evolution of the fraction of cells in the different cell classes for alcohol alone (Figure 7A), smoking alone (Figure 7B), and alcohol and smoking together (Figure 7C). In our simulations, alcohol alone does not cause a cancer field to develop (case A), while smoking alone and smoking with alcohol generates a cancer field (cases B and C). It would, however, be premature to conclude that alcohol has no effect on carcinogenesis. Alcohol causes cancer, but the mechanism might be different and not fully captured by the 10 genes that we consider here. Also, it is reported in Hashibe et al. (2009) and LoConte et al. (2018) that the carcinogenic effect of alcohol is significantly lower than the effect of smoking.

### 3.5. Tumor excision

In this section, we examine the dynamics of the field development after a tumor excision, including the recurrence time. We consider two types of excisions: (A) killing only the tumor and cancer stem cells but not the cancer field and (B) killing all mutated cell classes, including cancer and cancer field cells. Treatment schedule A corresponds to a targeted treatment such as an immunotherapy or an oncolytic viral therapy, while scenario B corresponds to a more global attack such as through radiation treatment or standard chemotherapy.

**Figure 8:**
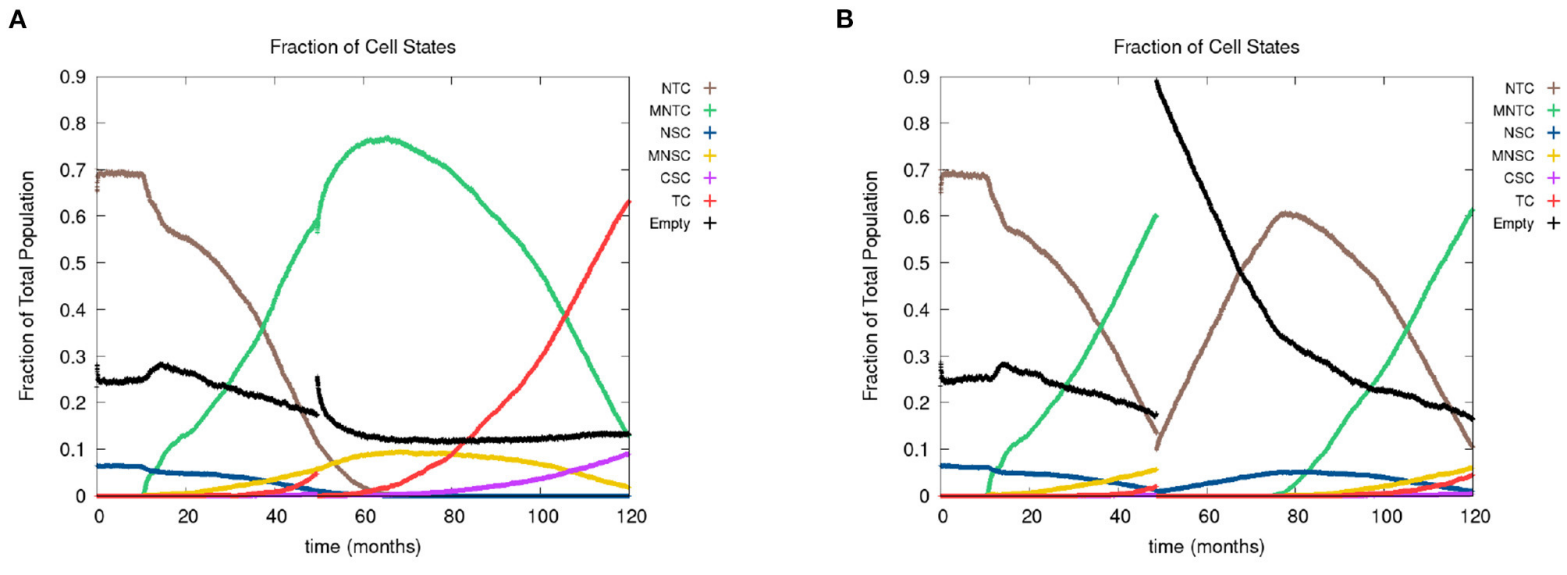
In figures **(A, B)**, we show the time course of the fraction of cells in the different cell classes NTC, MNTC, NSC, MNSC, CSC, TC, empty. In the plots, we consider the case where we **(A)** remove only TCs (keeping the field) and **(B)** we remove all mutated cells (removing the field). Parameters are as follows: grid size 256 × 256, both carcinogens activated, carcinogen spatial distribution 2, and time elapse of excision following first TC appearance was 18 months.

In Figure 8, we show the time evolution of the fraction of cells in the different cell classes. In Figure 8A, we consider the case of removing the cancer cells but not the cancer field cells and in Figure 8B, we show the case of removing the cancer and cancer field cells. Videos for the scenarios of keeping the field and removing the field are provided at https://youtu.be/zngGzjSlPwU and https://youtu.be/EOFI4Ai1A9U, respectively. Each video shows from left to right the carcinogen spatial distribution, the CA grid, and a visualization that shows the top 20 cell lineages. The excision occurs in the period of 40–60 months, prior to this period we observe normal cancer field and tumor development. We can follow the various sub populations in Figure 8. As the field develops, the number of normal tissue cells decreases as the number of mutated cells increases, with TC just starting to form and accelerate its growth and a very small uptake in CSCs beginning. At the point of excision there is a spike in the number of empty cells (black), which is more prominent in case B, and the number of tumor cells is set to be zero (red and purple). In the case B, the number of mutated cells (yellow) is reduced to zero as well. After an extended lag the field restarts its growth at about the same rate as originally. Whereas, when the field is kept intact (as in case A), the cancer comes back very quickly.

### 3.6. Cell lineages

**Figure 9:**
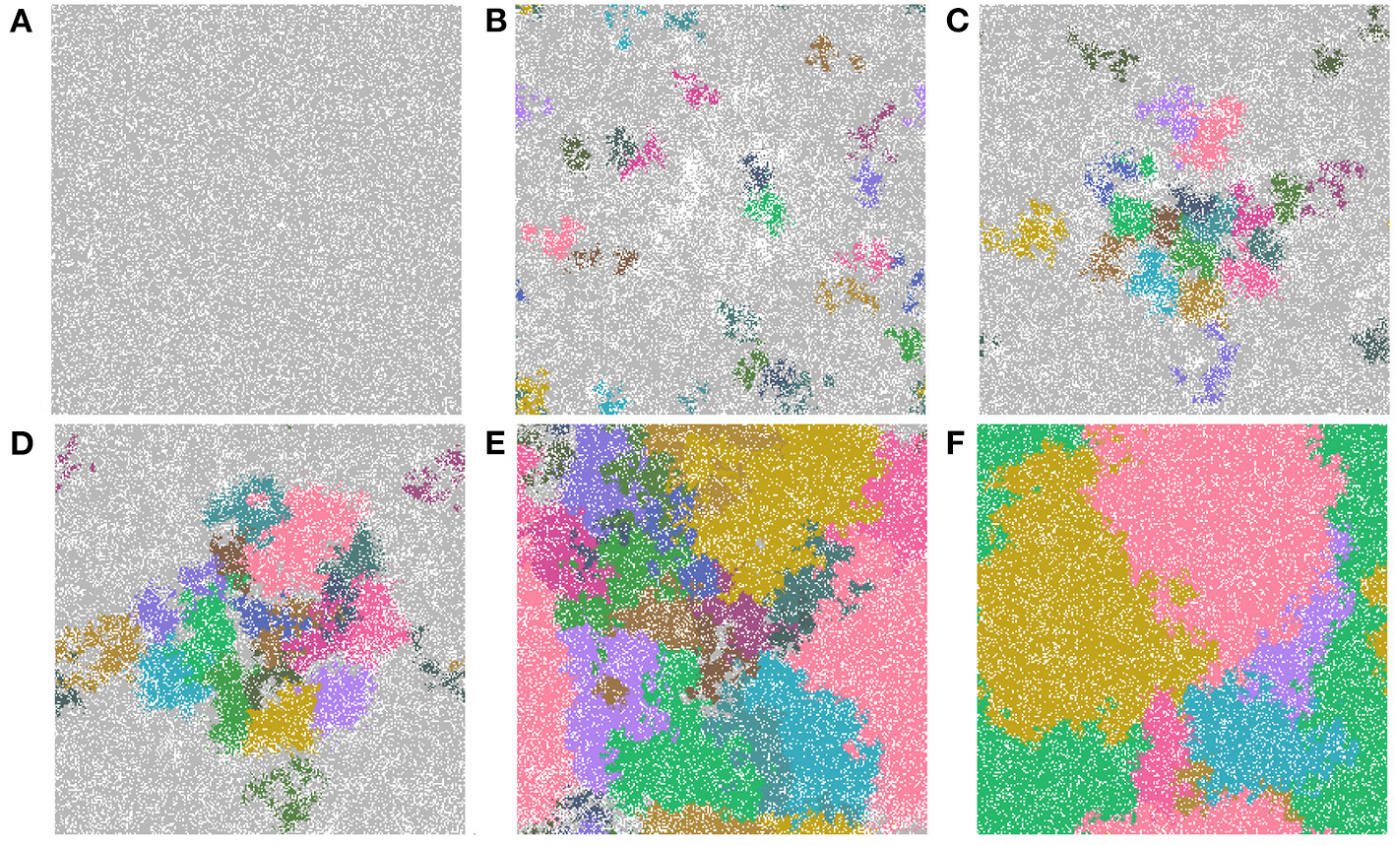
Important time-steps that show the top 20 lineages throughout the development stages of field cancerization. In the figures we show **(A)** the initial seed, **(B)** early cancer field formation, **(C)** later cancer field development, **(D–F)** cancer development. Note that light gray means the cell is not in any of the top 20 lineages and each color represents a different cell lineage. Parameters are as follows: grid size 256 × 256 and both carcinogens activated. See also video https://youtu.be/eKxsrSoDiKs.

The last feature that we include is an identification of cell lineages. In Figure 9, we show time-steps that are relevant for the major developmental stages of field cancerization. We display the top 20 cell lineages at each time step. The time steps include the initial seed (Figure 9A), the early development of the cancer field (Figures 9B, C), the first emergence of cancer cells (Figure 9D), full cancer development (Figure 9E) where all 20 leading lineages are cancer lineages, and finally a polyclonal tumor (Figure 9F) comprised of the fittest cancer lineages. The videos that are listed in Table 4 show the cell lineage developments over time. Figure 9 shows that the largest cell lineages in the domain are contained in the cancer field, which is expected because it contains the most fit cells in the domain.

In all experiments on the 256 × 256 grid where we followed the lineages we found polyclonal cancer fields. Monoclonal cancers were only observed at small grid sizes such as 64 × 64 and 128 × 128.

## 4. Conclusion

We developed a sophisticated cellular automata model for the cancer field effect. The model is an extension to existing cellular automata models (Gerlee and Anderson, 2007; Foo et al., 2014) as we include the effect of two carcinogens, one related to smoking and one related to alcohol. The impact on gene expression of oncogenes and tumor suppressor genes are of significant importance to the given cancer. Based on the existing literature we also find that smoking is a potent carcinogen, while the effect of alcohol is minimal. The gene expression was modeled by a multi-layer neural network, which can be trained once more data is available. We admit that our modeling of gene mutations and their impact on phenotype is simplified. To train the neural network for the gene expression dynamics, we would need single cell genomic data. These data need to explain the mutational changes in gene expression based on carcinogen exposure, plus an understanding of how mutated genes change the phenotypic actions of proliferation, apoptosis, quiescence and differentiation. The former could be obtained from large scale cell profiling. The second question, how do genes impact phenotype, is still an unresolved holy grail of genomics.

We demonstrated that when an excision is performed that removes only the tumor cells but leaves the remaining surrounding tissue intact, the cancer recurs faster than when removing the entire field of mutated tissue. When the field is not removed during excision, the cancer that recurs is more aggressive than before the field was removed. We observed, by tracking cell lineages, that the tumor masses mostly form from polyclonal origins.

There are a number of possible extensions to our model that might yield additional insights. A dynamic mutation threshold could be considered that depends on a number of factors such as the number of mutated genes or cell age. This mutation threshold could also be specific for each gene. For example, in our model we assumed TP53 is related to all other genes, and, as a result, once it is mutated, all other genes become mutated as well. However, it might be useful to consider a specific order of gene mutations that lead to cancer.

Telomeres are at the end of the DNA strands and with each cell division they get cut shorter, eventually becoming so short that the cell can no longer proliferate and so will enter senescence. Senescent cells are similar to quiescent cells except they can not perform any actions and eventually they undergo apoptosis. Therefore, the model could be enhanced by introducing telomeres.

It would be interesting to include viral infections to the model such as human papillomavirus (HPV) as input to the gene expression neural network (Lee and Tameru, 2012).

One of the questions we originally wanted to answer was how long it would take for a tumor to become large enough to be detected by physicians, however, we were not able to answer this question due to the size of the cells requiring at least a domain size 1024 × 1024 to represent the required 1*cm* detection size. A few simulations at 1024 × 1024 were run and we found it would take more than 10 years to fill in the space, thus it would take at least 10 years for the tumor to be detectable.

With regards to efficiency of running the model, as the complexity increases, the speed of the calculations involved in the gene expression neural network could be improved with linear algebra libraries available in CUDA. Using texture memory in the GPU to store cell neighborhoods would make calculations both faster and easier, as it has faster bandwidth and built in boundary conditions. The code could be made more cross compatible by allowing parallel computation on the CPU and switching from CUDA to OpenCL.

Finally, we note that there are many aspects of cancer biology that are not included here. Chemical signaling and feedback mechanisms among cancer cell sub populations are an important aspect of cancer growth, as well as the interactions with the immune response, mechanical aspects of the tissue, and angiogenesis. The seminal “hallmarks” papers of Hanahan and Weinberg (2000), Hanahan and Weinberg (2011), and Hanahan (2022) give a rather complete picture of effects that are important to cancer growth. As our model is already quite complicated, we did not include all of these effects here. But they are interesting extensions for future versions of the model.

We are grateful for the comments of two anonymous referees. One of them made the following interesting observation. People with a hereditary carcinogenic birth defect are predestined to form cancers, even early in their lives. For example, Rb mutations can lead to Retinoblastoma and the BRCA gene mutations can increase breast cancer risks. In such a case, would the entire body be considered a cancer field? This is an interesting question of further thought, which certainly exceeds the abilities of our computational model.

## Data availability statement

Publicly available datasets were analyzed in this study. The datasets can be accessed via the reference list.

## Author contributions

TH and JN led the study. KD developed the hybrid cellular automaton. TH, JN, and KD wrote the manuscript. All authors contributed to the article and approved the submitted version.
