# Supplementary material for: A computational model for the cancer field effect
Source: Front Artif Intell. 2023 Jul 4;6:1060879. doi: 10.3389/frai.2023.1060879 (PMC10352683; doi:10.3389/frai.2023.1060879)
Supplement: Supplementary file 1 [file Presentation_1.zip › frontiers2-supplement.pdf]

frontiers

A computational model for the cancer field effect - Supplemental Material

Karl Deutscher [1], **Thomas Hillen** [1] **and Jay Newby** [1]
[1] *University of Alberta, Edmonton, Alberta, Canada*
Correspondence*:
Thomas Hillen
thillen@ualberta.ca

1 FULL DESCRIPTION OF THE CELLULAR AUTOMATON

As mentioned in the model overview, the six cell classes that we consider are normal tissue cells (NTC), mutated normal tissue cells (MNTC), normal stem cells (NSC), mutated normal stem cells (MNSC), cancer stem cells (CSC), and tumour cells (TC), with numerical values 0 = NTC, 1 = MNTC, 2 = NSC, 3 = MNSC, 4 = CSC, 5 = TC. Since biological cells can move, proliferate, differentiate, and go through apoptosis then we must also introduce an empty cell class which is represented by the value 6. The cell class in the CA is represented by $s(t) \in \{0, 1, ..., 6\}$. Note that when visualizing the CA each value of $s(t)$ also has a colour associated to it.

Each cell in the CA tracks the gene expression of the G genes in a vector defined by

$$\boldsymbol{E}(t) = [\{e_j(t)\}_{j=1,...,G}]. \tag{1}$$

The phenotype of a cell is tracked by a vector that contains probabilities for each type of phenotypic action occurring in a given time-step and is defined by

$$\boldsymbol{P}(t) = [p(t), q(t), a(t), d(t)], \tag{2}$$

where $p(t)$ represents proliferation, $q(t)$ represents quiescence, $a(t)$ represents apoptosis, and $d(t)$ represents differentiation. The probabilities are set such that $\boldsymbol{P}(t)$ generates a probability distribution, so that

$$\sum_{i=1}^{4} P_i(t) \equiv p(t) + q(t) + a(t) + d(t) = 1 \text{ and } P_i(t) \geq 0, \forall t. \tag{3}$$

At a time-step in the CA a phenotypic action is chosen to occur by sampling from the probability distribution generated from $\boldsymbol{P}(t)$. Hence, since we do not want a cell to reproduce more than once in a time-step, each time-step represents the length of a typical cell cycle for the type of tissue under consideration.

When a NSC, MNSC, or CSC differentiate the resultant cell initially is a transit amplifying cell (TAC) for a set number of generations, Θ, after which it turns respectively into a NTC, MNTC, or TC. As a result of this each cell has two parameters $\overline{\tau}(t) \in \{0, 1\}$ and $\overline{n}(t) \in \{0, ..., \Theta\}$, where $\overline{\tau}(t)$ is a binary parameter used to determine if a cell is currently a TAC or not and $\overline{n}(t)$ is the number of generations a TAC cell lineage has produced. The parameters $\overline{\tau}(t)$ and $\overline{n}(t)$ are copied from parent to child cell and once $\overline{n}(t) = \Theta$ then $\overline{\tau}(t+1) = 0, \overline{n}(t+1) = 0$.

The final aspect of the cell that is tracked and represented in the overall cell state is the age of the cell, $\alpha(t) \in \mathbb{N}$. Taking all this information together, the state of a cell in the CA is given by the vector

$$\boldsymbol{S}(t) = [s(t), \alpha(t), \boldsymbol{E}(t), \boldsymbol{P}(t), \overline{\tau}(t), \overline{n}(t)] \tag{4}$$

The domain could theoretically be either two-dimensional or three-dimensional, however for simplicity and computational purposes, the domain considered will be two-dimensional and we employ periodic boundary conditions, i.e. the domain is a torus. Each cell has a neighbourhood that contains itself, the cardinal directions around it, and the cells directly NE, SE, SW, and NW of the cell. In CA theory this is called the Moore neighbourhood and is mathematically defined for two-dimensional grids as

$$N^M_{(x_0,y_0)} = \{(x,y) | |x - x_0| \leq r, |y - y_0| \leq r\}, \tag{5}$$

where r is the range of the Moore neighbourhood and (x_0, y_0) is the cell that the neighbourhood surrounds [2]. Most commonly the Moore neighbourhood used is with $r = 1$ and it is the one used here.

(A.1) Cell Mutation

A cell can become mutated through changes in both the likelihood of a phenotype occurring and the gene expression. The process of these changes is described in this section.

The chosen G genes are known genes related to the type of cancer being studied. Thus, each of the G genes is either a tumour suppressor gene or an oncogene. Define the vector $\boldsymbol{T} \in \{0,1\}^G$, where each element, T_j, represents gene j's type, whereby $T_j = 0$ represents a tumour suppressor gene and $T_j = 1$ represents an oncogene.

A gene j is positively mutated towards cancer (positively mutated) if it is mutated and either it is a tumour suppressor gene and its gene expression is downregulated, $T_j = 0$ and $e_j(t) \leq -\overline{M}$, or it is an oncogene and its gene expression is upregulated, $T_j = 1$ and $e_j(t) \geq \overline{M}$, where $\overline{M}$ is the given threshold.

At each time-step the gene expression of each gene is updated from the results of the gene expression neural network **??**. The changes in the gene expression allow the gene to become mutated or even go from mutated to non mutated (normally expressed). The following function is used as an indicator to determine if a gene j is mutated, positively mutated or normally expressed:

$$\Psi(t,j) = \begin{cases} 0, & |e_j(t)| < \overline{M} (\text{normally expressed}) \\ 1 - 2T_j, & e_j(t) \leq -\overline{M} (\text{underexpressed gene}) \\ -2 + 4T_j, & e_j(t) \geq \overline{M} (\text{overexpressed gene}) \end{cases} \tag{6}$$

Notice that when $\Psi(t,j) > 0$ the gene j is positively mutated and when $\Psi(t,j) < 0$ it is mutated away from cancer. Further take note that if $|\Psi(t,j)| = 1$ the gene j is downregulated and when $|\Psi(t,j)| = 2$ it is upregulated.

A given gene can influence the expression of another gene as follows. A positively mutated gene will cause a positive mutation of a related gene. A non-positively mutated gene will cause a negative mutation (mutation that regulates a gene towards normal expression) of a related gene. Define the matrix $R \in \{0,1\}^{G \times G}$, where each entry, R_{ij}, represents whether gene i is related to gene j with $0 =$ unrelated and $1 =$ related. Note that the matrix R is not necessarily symmetric as a gene i might regulate gene j but not vice versa. To update gene j according to formula (8) below, we chose a random number $z \sim U(0,1)$ and an update occurs only if $z \leq \overline{\gamma}$ and $R_{ij} = 1$. The process of

a gene i changing the gene expression of another gene j is represented by the formula:

$$\varsigma(t,i,j) = \begin{cases} 1, & (\Psi(t,i) > 0 \text{ and } T_j = 1 \text{ and } e_j(t) < M) \text{ or} \\ & (\Psi(t,i) \leq 0 \text{ and } e_j(t) < 0) \\ \\ -1, & (\Psi(t,i) > 0 \text{ and } T_j = 0 \text{ and } e_j(t) > -M) \text{ or} \\ & (\Psi(t,i) \leq 0 \text{ and } e_j(t) > 0) \\ \\ 0, & \text{otherwise} \end{cases}, \tag{7}$$

$$e_j(t) = e_j(t-1) + \varsigma(t-1,i,j)\varepsilon, \tag{8}$$

where $\varepsilon \sim U(\varepsilon_1, \varepsilon_2)$ is a randomly chosen increment between ε_1 and ε_2. The previous update is used by fixing a gene i and applying the function to all the other genes, then repeating the process on the next gene and so forth, until all the genes have been processed. In each of these steps new random numbers z, ε are generated.

In addition, if a gene j is mutated then there is a chance that the gene expression is negatively mutated, so to replicate the bodies attempting to revert mutated genes. The following function is applied only when some random variable $z \sim U(0,1)$ is less than or equal to a threshold $\overline{\phi}$. This is represented by the gene repair function:

$$e_j(t) = e_j(t-1) + \begin{cases} \varepsilon, & |\Psi(t-1,j)| = 1 \\ -\varepsilon, & |\Psi(t-1,j)| = 2 \end{cases}, \tag{9}$$

where $\varepsilon \sim U(\varepsilon_1, \varepsilon_2)$ is a random increment. In a given time-step the previous function (9) is applied once to all G genes.

(A.2) Update Rules for Phenotypic Action

Recall that the probabilities of phenotypic actions is given by

$$\boldsymbol{P}(t) = [p(t), q(t), a(t), d(t)], \tag{10}$$

where $p(t)$ represents proliferation, $q(t)$ represents quiescence, $a(t)$ represents apoptosis, and $d(t)$ represents differentiation.

We define the matrix $\overline{U} \in \mathbb{R}^{4\times G}$, where each entry, $\overline{U}_{ij}$, is an increment to the probability of phenotypic action i (defined by the above ordering), under the circumstance that gene j is mutated and its' expression is upregulated. Similarly, we define the matrix $\overline{D} \in \mathbb{R}^{4\times G}$, where each entry, $\overline{D}_{ij}$, is an increment to the probability of phenotype action i, under the circumstance that gene j is mutated and its' expression is downregulated.

These probabilities are updated at each time as follows. Let Q_t be the event of quiescence at time t and let its complement A_t be the event of an action $K_t \in \mathbb{A}$ where

$$\mathbb{A} = \{\text{differentiation, apoptosis, proliferation}\},$$

is the set of phenotypic actions. Hence, $P[Q_t] + P[A_t] = 1$ and $P[K_t \mid A_t]$ is the probability distribution over the three possible actions, given that an action occurs. The total probability of a given action $k \in \mathbb{A}$ is given by

$$\mathsf{P}_t(k) = P[K_t = k \mid A_t]P[A_t], \tag{11}$$

where

$$\sum_{k\in\mathbb{A}} \mathsf{P}_t(k) = P[A_t] = 1 - P[Q_t]. \tag{12}$$

Combining these, we can write the conditional probability in terms of the total probability with

$$P[K_t = k \mid A_t] = \frac{\mathsf{P}_t(k)}{\sum_{l\in\mathbb{A}} \mathsf{P}_t(l)}. \tag{13}$$

Note that in the notation of (10), we have that

$$q(t) = P[Q_t], \tag{14}$$

$$p(t) = \mathsf{P}_t(\text{proliferation}), \tag{15}$$

$$a(t) = \mathsf{P}_t(\text{apoptosis}), \tag{16}$$

$$d(t) = \mathsf{P}_t(\text{differentiation}). \tag{17}$$

When updating the probability of quiescence by a given increment Δ, we have

$$P[Q_t] = P[Q_{t-1}] + \Delta, \tag{18}$$

and then for each $k \in \mathbb{A}$, we set

$$\mathsf{P}_t(k) = P[K_{t-1} = k \mid A_{t-1}]P[A_t] = \frac{\mathsf{P}_{t-1}(k)}{\sum_{l\in\mathbb{A}} \mathsf{P}_{t-1}(l)}(1 - P[Q_t]). \tag{19}$$

The above yields an update formula for the probability of quiescence. Next, we derive a similar formula to update the phenotypic action probabilities $\mathsf{P}_t(k)$ given by (11). We assume that an update at time t to one of the three phenotypic action probabilities will not cause a corresponding change to the others. Instead, the probability of quiescence will be adjusted to add or remove the necessary probability from $P[A_t] = 1 - P[Q_t]$. For example, an increase in the probability of apoptosis will have a corresponding decrease in the probability of quiescence, but will not change the probabilities of differentiation and proliferation. To update a given probability of phenotypic action $k \in \mathbb{A}$ by a given increment $\tilde{\Delta}$ we use

$$\mathsf{P}_t(k) = \mathsf{P}_{t-1}(k) + \tilde{\Delta}, \quad P[A_t] = P[A_{t-1}] - \tilde{\Delta}. \tag{20}$$

Finally, the increment itself must be adjusted so that all updated probabilities remain bounded to the interval $(0, 1)$. Given the probability in the previous time step P_0 and the initially-proposed increment δ, the modified increment is given by

$$\Delta(\delta, P_0; \epsilon) = \begin{cases} -P_0(1-\epsilon), & P_0 + \delta \le 0 \\ (1-P_0)(1-\epsilon), & P_0 + \delta \ge 1 \\ \delta, & \text{otherwise} \end{cases}, \tag{21}$$

where $0 < \epsilon < 1$ is an adjustable parameter (we take $\epsilon = 10^{-2}$). With the above, we have that $P_{\text{new}} = P_0 + \Delta(\delta, P_0; \epsilon)$ will always be bounded such that $0 < P_{\text{new}} < 1$.

One final adjustment is necessary for the update formula (20). The update increment $\tilde{\Delta}$ must ensure that both $\mathsf{P}_t(k)$ and $P[Q_t]$ remain bounded to the interval $(0, 1)$. Let $\Delta_1 = \Delta(\delta, \mathsf{P}_{t-1}(k))$ and $\Delta_2 = \Delta(\delta, P[Q_{t-1}])$. We set

$$\tilde{\Delta} = \begin{cases} \Delta_1, & |\Delta_1| < |\Delta_2| \\ \Delta_2, & \text{otherwise} \end{cases}. \tag{22}$$

(A.3) Update Rules for Cell Class

The difference between the mutated class of a cell and the non-mutated class is that the mutated class has $\Upsilon \in \mathbb{N}$ positively mutated genes. Note that to delay the arrival of the first CSC not only does there have to be Υ positively mutated genes but for a SC or MNSC to transition into a CSC a random variable has to be less than the threshold $\iota \in \mathbb{R}_+(0, 1)$. This can be described by the following

$$\overline{\Psi}(t, j) = \begin{cases} 1, & \Psi(t, j) = 1 \text{ or } \Psi(t, j) = 2 \\ 0, & \text{otherwise} \end{cases}, \tag{23}$$

$$s(t) = \begin{cases} 0(\text{NTC}), & s(t-1) = 1(\text{MNTC}), \\ & \sum_{j=1}^{G} \overline{\Psi}(t, j) < \Upsilon \\ 1(\text{MNTC}), & s(t-1) = 0(\text{NTC}), \\ & \sum_{j=1}^{G} \overline{\Psi}(t, j) \geq \Upsilon \\ 2(\text{NSC}), & s(t-1) = 3(\text{MNSC}), \\ & \sum_{j=1}^{G} \overline{\Psi}(t, j) < \Upsilon \\ 3(\text{MNSC}), & s(t-1) = 2(\text{NSC}), \\ & \sum_{j=1}^{G} \overline{\Psi}(t, j) \geq \Upsilon \\ 4(\text{CSC}), & s(t-1) = 2(\text{NSC}), \\ & \sum_{j=1}^{G} \overline{\Psi}(t, j) \geq \Upsilon, \\ & z \leq \frac{\iota}{2} \\ 4(\text{CSC}), & s(t-1) = 3(\text{MNSC}), \\ & \sum_{j=1}^{G} \overline{\Psi}(t, j) \geq \Upsilon, \\ & z \leq \iota \end{cases}, \tag{24}$$

where $z \sim U(0, 1)$, $\Psi(t, j)$ is the mutation indicator function given by (6), and G is the number of genes.

Since each cell class has different probabilities for each phenotypic action, then there exists an initial phenotype matrix, $\tilde{P} \in \mathbb{R}^{6\times 4}(0, 1)$ with $\sum_{i=1}^{4} \tilde{P}_{k,i} = 1$, $k = 0, 1, 2, 3, 4, 5$. Therefore, whenever a non-empty cell changes

class, the phenotype vector must be set to its' new cell class's initial values, while also keeping all the changes that have happened to the phenotype vector. This is achieved through the following process

$$\varphi_i(t) = \tilde{P}_{k,i} + P_i(t-1) - \tilde{P}_{s(t-1),i} \tag{25}$$

where k is the new cell state, $i = 1, 2, 3, 4$.

Then, the probabilities are given by

$$P_i(t) = \frac{\varphi_i(t)}{\sum_{j=1}^{4} \varphi_j(t)}. \tag{26}$$

Dedifferentiation is the process of a specialized cell reverting back to a non-specialized cell. In our model this is accomplished by a non stem cell becoming a stem cell. Dedifferentiation is used to help maintain the proper ratio of stem cells to non stem cells in the grid by dedifferentiating whenever the number of stem cells in the neighbourhood of a non stem cell is less than or equal to some chosen value, $\hat{S}$, or if the number of empty cells in the neighbourhood of a non stem cell is less than or equal to some chosen value, $\hat{E}$. To help reduce the number of cells dedifferentiating, the process is completed only when a random sample from the uniform distribution is less than or equal to some threshold, $\hat{D} \in \mathbb{R}_+(0, 1)$. This process is represented by the function:

$$s(t) = \begin{cases} 2(\text{NSC}), & s(t-1) = 0(\text{NTC}) \\ 3(\text{MNSC}), & s(t-1) = 1(\text{MNTC}) \\ 4(\text{CSC}), & s(t-1) = 5(\text{TC}) \end{cases}. \tag{27}$$

(A.4) Cell Fitness

Each cell has a fitness value associated to it, so that the cells can compete and the population contains only the healthiest, or in the case of mutated cells, the most positively mutated cells. The characteristics that affect the fitness are based upon work by [1], in which they point to the following important characteristics:

1. if a cell has a high apoptotic rate, it is less fit;
2. if a cell has a high proliferation rate, it is more fit;
3. if a cell is older, then it is less fit;
4. if a gene in a cell is mutated towards cancer, than it is less fit unless the cell is cancerous, in which case it is more fit;
5. if a cell is what they call super-competitive, then it is more fit than any type of cell.

Based upon this the fitness of a cell in the CA is computed using the functions $\hat{M}$ which is the gene expression ratio and the fitness function $\hat{F}$:

$$\hat{M}(t,j) = \begin{cases} -\dfrac{E_j(t)}{\overline{M}} & ,(T_j = 0 \text{ and } E_j(t) < 0) \\ & \text{or } (T_j = 1 \text{ and } E_j(t) > 0) \\ \dfrac{E_j(t)}{\overline{M}} & ,(T_j = 0 \text{ and } E_j(t) > 0) \\ & \text{or } (T_j = 1 \text{ and } E_j(t) < 0) \\ 0 & ,\text{otherwise} \end{cases}, \tag{28}$$

$$\begin{aligned} \hat{F}(t) = & \begin{cases} \dfrac{P_1(t)}{\tilde{P}_{s(t)1}} & ,\overline{\tau}(t) = 0 \\ \dfrac{P_1(t)}{\tilde{P}_{s(t)1} + \omega} & ,\overline{\tau}(t) = 1 \end{cases} \\ & - \frac{P_3(t)}{\tilde{P}_{s(t)3}} - \alpha(t)P_3(t) \\ & + \begin{cases} \sum_{j=1}^{G} \hat{M}(t,j) & ,s(t) = 0 \text{ (NTC) or } s(t) = 2 \text{(NSC)} \\ -\sum_{j=1}^{G} \hat{M}(t,j) & ,\text{otherwise} \end{cases}, \end{aligned} \tag{29}$$

where $E_j(t)$ is the gene-expression of gene j, $\overline{M}$ is the threshold that determines where gene j is mutated, T_j indicates if the gene j is either a tumour suppressor gene or oncogene, $P_1(t)$ is the probability that proliferation will occur in a time-step, $P_3(t)$ is the probability that apoptosis will occur in a time-step, $\alpha(t)$ is the age of the cell, $\overline{\tau}(t)$ indicates if the cell is a TAC or not, and $\overline{P}$ is the initial phenotype matrix that provides the initial values for each of the phenotypic actions for each cell type. If the cell is from a SC class (SC, MNSC, CSC) then its' fitness is multiplied by a factor $\Pi \in \mathbb{R}_+$ if $\hat{F}(t) > 0$ or Π^{-1} if $\hat{F}(t) < 0$, so that a SC type cell has a higher fitness than a non-stem cell.

(A.5) CA Rule

Each non-empty cell in the CA grid chooses a phenotypic action to execute for that time-step and attempts to complete such action. The phenotypic action is chosen by taking a random sample from the probability distribution that is generated by the phenotype vector. Consider that the cell that is performing the phenotypic action is located at $\boldsymbol{x}^{(p)} \in \Omega$. The cell the action is being applied to will be located at $\boldsymbol{x}^{(c)} \in \Omega$, which is a randomly chosen location in the cell's neighbourhood. The randomly chosen cell is an empty cell or a cell with a lower fitness in the case of proliferation, and differentiation. It is important to note that only NSC, MNSC, and CSC can differentiate.

CSCs and TCs are the only class of cells that can kill other cells when moving during quiescence. If the parent cell is a CSC or TC and the chosen cell has a higher fitness then the phenotypic action is accomplished only if a sample from some random variable is less than a threshold to kill, $\kappa \in \mathbb{R}_+(0,1)$. A CSC can kill a TC and TC a CSC only if

the fitness is lower, as otherwise the tumour cell population and/or CSC population would die out before they have had the chance to thrive.

Assuming all the conditions for the phenotypic action to occur are met then the following changes occur to the states of the parent and child cell upon **proliferation**:

$$\boldsymbol{S}(\boldsymbol{x}^{(k)},t)=\begin{cases}[s(\boldsymbol{x}^{(p)},t-1),0,\boldsymbol{E}(\boldsymbol{x}^{(p)},t-1),\boldsymbol{P}(\boldsymbol{x}^{(p)},t-1), & ,\overline{\tau}(\boldsymbol{x}^{(p)},t-1)=0\\ \quad\overline{\tau}(\boldsymbol{x}^{(p)},t-1),\overline{n}(\boldsymbol{x}^{(p)},t-1)] & \\ & \\ [s(\boldsymbol{x}^{(p)},t-1),0,\boldsymbol{E}(\boldsymbol{x}^{(p)},t-1),\boldsymbol{P}(\boldsymbol{x}^{(p)},t-1), & ,\overline{\tau}(\boldsymbol{x}^{(p)},t-1)=1,\\ \quad\overline{\tau}(\boldsymbol{x}^{(p)},t-1),\overline{n}(\boldsymbol{x}^{(p)},t-1)+1] & \overline{n}(\boldsymbol{x}^{(p)},t-1)\neq\Theta\\ & \\ [s(\boldsymbol{x}^{(p)},t-1),0,\boldsymbol{E}(\boldsymbol{x}^{(p)},t-1), & ,\overline{\tau}(\boldsymbol{x}^{(p)},t-1)=1,\\ \quad[P_1(\boldsymbol{x}^{(p)},t-1)-\Delta(\omega,P_1(\boldsymbol{x}^{(p)},t-1)), & \\ \quad P_2(\boldsymbol{x}^{(p)},t-1)+\Delta(\omega,P_2(\boldsymbol{x}^{(p)},t-1)) & \overline{n}(\boldsymbol{x}^{(p)},t-1)=\Theta\\ \quad P_3(\boldsymbol{x}^{(p)},t-1),P_4(\boldsymbol{x}^{(p)},t-1)],0,0] & \end{cases}, \tag{30}$$

where $k\in\{p,c\}$; $\omega\in\mathbb{R}_+(0,1)$; $\boldsymbol{S}(\cdot,t)$ is defined by equation (4); Δ, defined by equation (21), ensures that the increment ω does not cause the phenotypic action being modified to leave the interval $[0,1]$.

Assuming all the conditions for the phenotypic action to occur are met then the following changes occur to the states of the parent and child cell upon **cell movement**:

$$\boldsymbol{S}(\boldsymbol{x}^{(p)},t)=\begin{cases}[6,0,\mathbf{0},\mathbf{0},0,0] & ,z<\zeta\\ & \\ [s(\boldsymbol{x}^{(p)},t-1),\alpha(\boldsymbol{x}^{(p)},t-1),\boldsymbol{E}(\boldsymbol{x}^{(p)},t-1), & ,\text{otherwise}\\ \quad\boldsymbol{P}(\boldsymbol{x}^{(p)},t-1),\overline{\tau}(\boldsymbol{x}^{(p)},t-1),\overline{n}(\boldsymbol{x}^{(p)},t-1)] & \end{cases}, \tag{31}$$

$$\boldsymbol{S}(\boldsymbol{x}^{(c)},t)=\begin{cases}[s(\boldsymbol{x}^{(p)},t-1),\alpha(\boldsymbol{x}^{(p)},t-1),\boldsymbol{E}(\boldsymbol{x}^{(p)},t-1), & ,z<\zeta\\ \quad\boldsymbol{P}(\boldsymbol{x}^{(p)},t-1),\overline{\tau}(x_1,y_1,t-1,\overline{n}(\boldsymbol{x}^{(p)},t-1)] & \\ & \\ [s(\boldsymbol{x}^{(c)},t-1),\alpha(\boldsymbol{x}^{(c)},t-1),\boldsymbol{E}(\boldsymbol{x}^{(c)},t-1), & ,\text{otherwise}\\ \quad\boldsymbol{P}(\boldsymbol{x}^{(c)},t-1),\overline{\tau}(\boldsymbol{x}^{(c)},t-1),\overline{n}(\boldsymbol{x}^{(c)},t-1)] & \end{cases}, \tag{32}$$

where $z\sim U(0,1)$ and $\zeta\in\mathbb{R}_+(0,1)$ is the threshold that must be met for the cell to move.

The following changes occur to the state of the cell at $\boldsymbol{x}^{(p)}$ upon **apoptosis**:

$$\boldsymbol{S}(\boldsymbol{x}^{(p)},t)=[6,0,\mathbf{0},\mathbf{0},0,0]. \tag{33}$$

Assuming all the conditions for the phenotypic action to occur are met then the following changes occur to the states of the parent and child cell upon **differentiation**:

$$\begin{aligned}\boldsymbol{S}(\boldsymbol{x}^{(p)},t) = [&s(\boldsymbol{x}^{(p)},t-1),0,\boldsymbol{E}(\boldsymbol{x}^{(p)},t-1),\\ &\boldsymbol{P}(\boldsymbol{x}^{(p)},t-1),\overline{\tau}(\boldsymbol{x}^{(p)},t-1),\overline{n}(\boldsymbol{x}^{(p)},t-1)]\end{aligned} \tag{34}$$

$$\boldsymbol{S}(\boldsymbol{x}^{(c)},t) = \begin{cases} \begin{aligned}&[0(\text{NTC}),0,\boldsymbol{E}(\boldsymbol{x}^{(p)},t-1),\\ &[P_1(\boldsymbol{x}^{(p)},t-1)\\ &\quad+\Delta(\omega,P_1(\boldsymbol{x}^{(p)},t-1)),\\ &\ P_2(\boldsymbol{x}^{(p)},t-1)\\ &\quad-\Delta(\omega,P_2(\boldsymbol{x}^{(p)},t-1)),\\ &\ P_3(\boldsymbol{x}^{(p)},t-1),P_4(\boldsymbol{x}^{(p)},t-1)],\\ &1,0]\end{aligned} & ,s(\boldsymbol{x}^{(p)},t-1)=2(\text{NSC}) \\ \\ \begin{aligned}&[1(\text{MNTC}),0,\boldsymbol{E}(\boldsymbol{x}^{(p)},t-1),\\ &[P_1(\boldsymbol{x}^{(p)},t-1)\\ &\quad+\Delta(\omega,P_1(\boldsymbol{x}^{(p)},t-1)),\\ &\ P_2(\boldsymbol{x}^{(p)},t-1)\\ &\quad-\Delta(\omega,P_2(\boldsymbol{x}^{(p)},t-1)),\\ &\ P_3(\boldsymbol{x}^{(p)},t-1),P_4(\boldsymbol{x}^{(p)},t-1)],\\ &1,0]\end{aligned} & ,s(\boldsymbol{x}^{(p)},t-1)=3(\text{MNSC}) \\ \\ \begin{aligned}&[5(\text{TC}),0,\boldsymbol{E}(\boldsymbol{x}^{(p)},t-1),\\ &[P_1(\boldsymbol{x}^{(p)},t-1)\\ &\quad+\Delta(\omega,P_1(\boldsymbol{x}^{(p)},t-1)),\\ &\ P_2(\boldsymbol{x}^{(p)},t-1)\\ &\quad-\Delta(\omega,P_2(\boldsymbol{x}^{(p)},t-1)),\\ &\ P_3(\boldsymbol{x}^{(p)},t-1),P_4(\boldsymbol{x}^{(p)},t-1)],\\ &1,0]\end{aligned} & ,s(\boldsymbol{x}^{(p)},t-1)=4(\text{CSC}) \end{cases}, \tag{35}$$

where $\omega \in \mathbb{R}_+(0,1)$ is the amount the probability of proliferation changes when the cell is a TAC.

(A.6) Lineage Tracking

The lineage of each cell is tracked for the purpose of following tumour cell lineages from their origin, checking how many independent tumour masses form throughout the simulation, and whether the origin is monoclonal or polyclonal. The lineage is tracked using the following methods. Each cell has a parameter $\Lambda(t) \in \{-1,0,1,...,LM-1\}$, where L is the length in the x-direction and M is the length in the y-direction, that when set to -1 implies no information is known or the cell is empty, and if set to any other value represents the spatial index of the parent at the time it created the first cell in the lineage.

During cell movement the parameter is passed down from parent ($\boldsymbol{x}^{(p)}$) to child cell ($\boldsymbol{x}^{(c)}$). In the case of apoptosis the parameter is set to -1. Finally, during proliferation and differentiation the parameter is set as follows for the

cells $\boldsymbol{x}^{(p)}$ and $\boldsymbol{x}^{(c)}$:

$$\Lambda(t) = \begin{cases} x_1^{(p)} M + x_2^{(p)}, & \Lambda(\boldsymbol{x}^{(p)}, t-1) = -1 \\ \Lambda(\boldsymbol{x}^{(p)}, t-1), & \text{otherwise} \end{cases}. \tag{36}$$

(37)

REFERENCES

[1]Sarah Bowling, Katerina Lawlor, and Tristan A. Rodríguez. Cell competition: the winners and losers of fitness selection. *Development*, 146(13):1–12, 2019.

[2]Lawrence Gray. A mathematician looks at Wolfram's new kind of science. *Notices of the American Mathematical Society*, 50(2):200–211, 2003.
